# Alpha-SNAP (M105I) mutation promotes neuronal differentiation of neural stem/progenitor cells through overactivation of AMPK

**DOI:** 10.3389/fcell.2023.1061777

**Published:** 2023-04-11

**Authors:** Felipe A. Bustamante-Barrientos, Maxs Méndez-Ruette, Luis Molina, Tania Koning, Pamela Ehrenfeld, Carlos B. González, Ursula Wyneken, Roberto Henzi, Luis Federico Bátiz

**Affiliations:** ^1^ Immunology Program, Centro de Investigación e Innovación Biomédica (CiiB), Universidad de los Andes, Santiago, Chile; ^2^ Neuroscience Program, Centro de Investigación e Innovación Biomédica (CiiB), Universidad de los Andes, Santiago, Chile; ^3^ PhD Program in Biomedicine, Facultad de Medicina, Universidad de los Andes, Santiago, Chile; ^4^ IMPACT, Center of Interventional Medicine for Precision and Advanced Cellular Therapy, Santiago, Chile; ^5^ Facultad de Medicina y Ciencia, Universidad San Sebastián, Puerto Montt, Chile; ^6^ Instituto de Inmunología, Facultad de Medicina, Universidad Austral de Chile, Valdivia, Chile; ^7^ Laboratory of Cellular Pathology, Institute of Anatomy, Histology and Pathology, Faculty of Medicine, Universidad Austral de Chile, Valdivia, Chile; ^8^ Center for Interdisciplinary Studies on Nervous System (CISNe), Universidad Austral de Chile, Valdivia, Chile; ^9^ Instituto de Fisiología, Facultad de Medicina, Universidad Austral de Chile, Valdivia, Chile; ^10^ School of Medicine, Facultad de Medicina, Universidad de Los Andes, Santiago, Chile; ^11^ Laboratorio de Reproducción Animal, Escuela de Medicina Veterinaria, Facultad de Recursos Naturales, Universidad Católica de Temuco, Temuco, Chile

**Keywords:** neurogenesis, hydrocephalus with hop gait, brain development, cell metabolism, cell fate, AMPK phosphatase, proliferation, ventricular zone

## Abstract

**Background:** The M105I point mutation in α-SNAP (Soluble N-ethylmaleimide-sensitive factor attachment protein-alpha) leads in mice to a complex phenotype known as hyh (hydrocephalus with hop gait), characterized by cortical malformation and hydrocephalus, among other neuropathological features. Studies performed by our laboratory and others support that the hyh phenotype is triggered by a primary alteration in embryonic neural stem/progenitor cells (NSPCs) that leads to a disruption of the ventricular and subventricular zones (VZ/SVZ) during the neurogenic period. Besides the canonical role of α-SNAP in SNARE-mediated intracellular membrane fusion dynamics, it also negatively modulates AMP-activated protein kinase (AMPK) activity. AMPK is a conserved metabolic sensor associated with the proliferation/differentiation balance in NSPCs.

**Methods:** Brain samples from hyh mutant mice (hydrocephalus with hop gait) (B6C3Fe-a/a-Napahyh/J) were analyzed by light microscopy, immunofluorescence, and Western blot at different developmental stages. In addition, NSPCs derived from WT and hyh mutant mice were cultured as neurospheres for *in vitro* characterization and pharmacological assays. BrdU labeling was used to assess proliferative activity *in situ* and *in vitro*. Pharmacological modulation of AMPK was performed using Compound C (AMPK inhibitor) and AICAR (AMPK activator).

**Results:** α-SNAP was preferentially expressed in the brain, showing variations in the levels of α-SNAP protein in different brain regions and developmental stages. NSPCs from hyh mice (hyh-NSPCs) displayed reduced levels of α-SNAP and increased levels of phosphorylated AMPKα (pAMPKα^Thr172^), which were associated with a reduction in their proliferative activity and a preferential commitment with the neuronal lineage. Interestingly, pharmacological inhibition of AMPK in hyh-NSPCs increased proliferative activity and completely abolished the increased generation of neurons. Conversely, AICAR-mediated activation of AMPK in WT-NSPCs reduced proliferation and boosted neuronal differentiation.

**Discussion:** Our findings support that α-SNAP regulates AMPK signaling in NSPCs, further modulating their neurogenic capacity. The naturally occurring M105I mutation of α-SNAP provokes an AMPK overactivation in NSPCs, thus connecting the α-SNAP/AMPK axis with the etiopathogenesis and neuropathology of the hyh phenotype.

## 1 Introduction

Radial glial cells (RGCs), also known as neural stem/progenitor cells (NSPCs), play critical roles in the formation of the central nervous system (CNS). They give rise to most neurons and macroglial cells, and function as railways for instructing neuronal radial migration (Rakic, 1972; Bustamante et al., 2019). In rodents, most RGCs are located in the ventricular zone (VZ), extending short apical and long basal processes (4, 5). This polarized structure allows symmetric and asymmetric cell divisions, balancing proliferation, self-renewing, and differentiation (Hartfuss et al., 2001; Noctor et al., 2002; Gotz and Huttner, 2005). The temporal and regional coordination between proliferation and differentiation of NSPCs during embryonic brain development depends on a delicate and regulated balance between extrinsic signals and intrinsic pathways (Gotz and Huttner, 2005). Defects in this biological balance have been associated with primary alterations in the neurogenic process, leading to the onset and progression of various brain developmental disorders, including microcephaly, hydrocephalus, and epilepsy (Rodriguez and Guerra, 2017; Yoon et al., 2017; Thodeson et al., 2018; Bustamante et al., 2019).

AMP-activated protein kinase (AMPK) is a metabolic sensor broadly conserved across eukaryotes, which functions as a central regulator of the bioenergetic status and metabolic transition from cell anabolism to catabolism (Garcia and Shaw, 2017). Either oscillations in AMP:ATP and ADP:ATP ratios and glycogen availability are constantly sensed by AMPK through substrate-specific binding domains, allowing cells to adapt and generate energy when it is limited (Garcia and Shaw, 2017). This metabolic sensing mechanism represents an essential support in the biology of different types of stem cells, including NSPCs (Zang et al., 2008; Zang et al., 2009). Previous studies have demonstrated that AMPK activity modulates the proliferation/differentiation balance in embryonic and adult NSPCs (Dasgupta and Milbrandt, 2009; Liang et al., 2018). Thus, abnormal regulation of AMPK activity could contribute to the onset and progression of distinct brain developmental disorders.

Soluble N-ethylmaleimide-sensitive factor (NSF) attachment protein alpha (α-SNAP) is a multifunctional protein associated with the fine regulation of intracellular traffic dynamics by controlling the zippering and disaggregation of SNAP receptors (SNAREs) (Barnard et al., 1997; Park et al., 2014). In addition to this canonical NSF-dependent function, α-SNAP participates in various NSF- and SNAREs-independent (non-canonical) physiological processes, such as autophagy induction (Naydenov et al., 2012b), apoptosis regulation (Naydenov et al., 2012a), calcium channels activity (Miao et al., 2013), and AMPK signaling (Wang and Brautigan, 2013). Indeed, *in vitro* experiments have demonstrated that α-SNAP can negatively regulate AMPK activity by acting as a relatively specific AMPK-phosphatase, reducing the phosphorylation of its alpha subunit at threonine 172 (pAMPKα^Thr172^) under metabolic stress conditions (Wang and Brautigan, 2013).

Since the deletion of the α-SNAP gene (Napa) generates early embryonic lethality (Chae et al., 2004), the role of α-SNAP on AMPK activity in NSPCs during brain development represents an unresolved challenge. The naturally occurring M105I mutation in α-SNAP protein, also known as hydrocephalus with hop gait (hyh) mutation, provokes a neurodevelopmental phenotype characterized by abnormal neurogenesis, cortical maldevelopment, agenesia of the corpus callosum, and cerebellar hypoplasia, among other features (Chae et al., 2004; Hong et al., 2004; Batiz et al., 2006; Ferland et al., 2009). *In vitro* studies suggest that, under energy stress conditions, overexpression of mutant α-SNAP (M105I) inhibits AMPK activity more efficiently than α-SNAP wild-type (Wang and Brautigan, 2013). However, when the M105I mutation naturally occurs in hyh mice, it provokes a reduction of α-SNAP protein levels (hypomorphism) (Chae et al., 2004; Arcos et al., 2017); thus, reducing its function (Chae et al., 2004; Hong et al., 2004).

This work aimed to study the consequences of naturally occurring α-SNAP M105I mutation on AMPK activity in NSPCs and its possible impact on their neurogenic capacity. We found that NSPCs derived from hyh mice show preferential commitment with the neuronal lineage and increased AMPK activity. Remarkably, pharmacological inhibition of AMPK generated a dramatic suppression of neuronal overproduction in hyh NSPCs, suggesting that AMPK overactivation is mediating at least partially the abnormal cell fate of hyh-NSPCs.

## 2 Materials and methods

### 2.1 Animals

The hyh mutant mice (hydrocephalus with hop gait) (B6C3Fe-a/a-Napa^*hyh*^/J) were obtained from The Jackson Laboratory (Bar Harbor, ME) and maintained at the animal facilities of the Universidad Austral de Chile, Valdivia, Chile and Universidad de los Andes, Santiago, Chile. Mice were maintained under a constant 12 h light/12 h dark photoperiod with a constant room temperature of ∼24°C. The embryonic day (E) 0.5 was based on the presence of a vaginal plug in the pregnant dam. Animals at E10.5, E12.5, E14.5, E16.5, E18.5, and newborns (postnatal day (PN) 1, 5) were used for different experiments. WT and homozygous mutant hyh embryos/pups were identified using a PCR-based genotyping method described by Batiz et al. (2009b).

### 2.2 Western blotting

Western blot analyses were performed as described previously (Batiz et al., 2009a; Arcos et al., 2017). Briefly, tissue samples or neurospheres were homogenized in modified RIPA buffer containing 50 mM Tris-HCl [pH 7.4], 150 mM NaCl, 1% NP-40, 1 mM EGTA, 1 mM EDTA, supplemented with 1mM PMSF, 2 mM AEBSF, 130 µM bestatin, 14 μM E−64, 1 µM leupeptin, 0.3 µM aprotinin, 1 mM Na3VO4, 50 mM NAF, 10 mg/mL trypsin inhibitor, and 10 mM sodium pyrophosphate (all from Sigma, St. Louis, MO). Equal amounts of total proteins were subjected to SDS-PAGE in 12% polyacrylamide gels and then electrotransferred onto PVDF membranes (Merck Millipore, Burlington, MA United States). Later, membranes were incubated for 1.5 h at 37°C with primary antibodies against: phospho-AMPKα-Thr172 (1:1,000), AMPKα (1:1,000), phospho-ACC-Ser79 (1:2,000), ACC (1:2000), phospho-TSC2-Ser1387 (1:2,000), all from Cell Signaling Technologies Denver; α-SNAP (1:1,000, 4E4; Exalpha Biological Inc., Maynard), βIII-tubulin (1:1,000; Promega Corp.), Nestin (1:2000; Merck), and GFAP (1:1,000; Dako Agilent, Santa Clara). β-Actin (DSHB, Iowa, US) and GAPDH (Merck S.A., Darmstadt, DE) were used as loading controls. Secondary antibodies were incubated by 1 h at RT and then detected by a chemiluminescence kit (Immobilon HRP, Merck S.A., Darmstadt, DE) using UVP ChemStudio Series- Western blot Imagers (Analytik Jena, LLC). The antibodies were diluted in PBS containing 0.35% Tween 20% and 5% BSA. In some cases, the membranes were stripped at RT with 62.5 mM Tris-HCl [pH 6.8], 2% SDS, and 0.7% β-mercaptoethanol for 30 min.

### 2.3 Light microscopy and immunofluorescence

Brain and neurospheres (NS) from WT and hyh mice were processed for histological analyses as previously described (Ferland et al., 2009). Briefly, animal brains and NS were fixed by immersion in Bouin’s solution or 4% paraformaldehyde for 48 h (brain samples) or 24 h (NS) at room temperature. Then, samples were dehydrated and included in Paraplast^®^. Histological sections were obtained and mounted on slides treated with silane (3-amino-propyl-triethoxy-silane) (Polysciences Inc., United States).

### 2.4 Light microscopy

For this purpose, the samples were stained with Hematoxylin/Eosin and analyzed under an AxioScope light microscope (Carl Zeiss, Jena, Germany)

### 2.5 Immunofluorescence

The primary antibodies used were mouse anti-Nestin (1:20 (DSHB, Iowa), mouse anti-βIII-tubulin (1:500; Promega Corp., Wisconsin), rabbit anti-GFAP (1:750 (Dako Agilent, Santa Clara, United States), rabbit anti-pAMPK (1:50 (Cell signaling, Denver, United States), mouse anti-α-SNAP (1:50, Exalpha Biological Inc., Maynard). All antibodies were diluted in a solution containing 0.7% carrageenan and 0.1% Triton-X 100 in 42.5 mM Tris-HCl pH 7.8 (all from Sigma, St. Louis, MO). In some cases, antigen retrieval with buffer citrate pH 6,0 in a microwave oven was performed. Serial sections were studied under a Leica SP8 confocal microscope using the acquisition software Leica Application Suite X Life Science (Leica Microsystems, Wetzlar, DE). Four different coronal sections per animal (n = 3 animals per condition), separated by at least 200 μm, were used to count the number of BrdU + cells and βIII-tubulin + cells in the VZ/SVZ, and to quantify Nestin and pAMPK fluorescence intensity. For quantification of βIII-tubulin + cells, three regions of interest (ROIs) of 14,000 μm^2^ (89 µm width, 157 µm length) were considered in each section. For quantification of Nestin and pAMPK fluorescence intensity, one region of interest (ROI) of 14,000 μm^2^ (80 µm length from ventricular surface, 175 µm width) was considered in each section. For quantification of pAMPK fluorescence intensity, one ROI of 2,000 μm^2^ (20 µm length from ventricular surface, 100 µm width) was considered in each section. Morphological changes of the Soma of Nestin + individual cells (located in the VZ) were analyzed in Nestin immunostained sections by calculating a cell elongation index (EI) according to (Park et al., 2018). The EI was expressed as a percentage and calculated as EI=(AB–L)/(AB + L), where AB and L represent the apical-basal (major) and lateral (minor) axes of the ellipse-shaped NSPCs Soma. Cell counts and fluorescence intensity (integrated density per area = mean gray value. Pixel number) were quantified using the Fiji open-source platform for biological-image analysis (Schindelin et al., 2012).

### 2.6 5-Bromo-2-deoxyuridine (BrdU) labeling in embryonic brains

BrdU is a thymidine analog that can be incorporated into newly synthesized DNA strands during S phase of cycling cells, and then be detected by immunocytochemistry using an anti-BrdU primary antibody. Pregnant dams at E14.5 were injected intraperitoneally with a single dose of BrdU (100 mg/kg) and euthanized 2 h later. BrdU incorporation in VZ/SVZ proliferating cells was detected by immunofluorescence using anti-BrdU (1:50; DSHB, United States). For quantification of BrdU-positive cells, a ventricular surface of 300 µm length was considered in each section. At least, four sections were analyzed per animal.

### 2.7 Neurosphere assay and monolayer culture of NSPCs

This procedure was performed as described by Guerra et al. (2015) with minor modifications (Guerra et al., 2015). Briefly, NSPCs were isolated from the dorsal telencephalon of WT and hyh mice and then cultured in NeuroCult basal medium, supplemented with NeuroCult proliferation medium, 20 ng/mL epidermal growth factor, 2 μg/mL heparin (StemCell Technologies, Vancouver, CA) and 100 μg/mL penicillin/streptomycin (Sigma, St. Louis). Viable cells were counted by the trypan blue cell viability assay and seeded in a non-adherent plate dish (Thermo Fisher Scientific, Waltham) to a cell density of 60,000 cells/mL. The cells were passaged three times and cultured for 7 days *in vitro* (DIV) in their last passage before every experiment. In some experiments, neurospheres of three passages were mechanically disaggregated and seeded as individual cells over poly-l-lysine coverslips and cultured for three additional DIV.

### 2.8 Measurement of ATP levels

Cellular ATP content was measured using the Cell Titer-Glo Luminescent Cell Viability Assay (Promega G7570, United States) according to manufacturer’s protocol. This assay generates a luminescent signal directly proportional to the amount of ATP in metabolically active cultured cells. Briefly, NSPCs isolated from WT and hyh mutant mice were cultured as described in 2.7 until the third passage and then plated in an opaque-walled 96-well-plate at a density of 5.10^4^ cells per well (in 100 µL of the media). Then, 100 µL of the Cell Titer-Glo reagent was added to each well. The plate was incubated for 10 min at room temperature. Luminescence was recorded in a BioTek FLx800 microplate reader and data were presented graphically as relative luminescence units (RLU).

### 2.9 BrdU incorporation assay in neurospheres

After 6 days *in vitro* (DIV), neurospheres were exposed to 20 mmol/L BrdU during a 24-h period. After treatment, neurospheres were collected by centrifugation, fixed, and embedded in paraffin. Serial sections were obtained, immunostained with anti-BrdU (1:200; DSHB, United States), and counterstained with Hematoxylin. Immunoreaction was detected using Elite Vectastain ABC kit (PK-6200, Vector Labs, United States). BrdU labeling index (i.e., the fraction of BrdU-positive cells over the total number of cells) was calculated in at least three sections per neurosphere and five neurospheres per animal.

### 2.10 Neurosphere differentiation assay

This was performed as described by Jimenez et al. (2009) (Jimenez et al., 2009), with some modifications. Briefly**,** in their third passage and after 7 DIV, ∼20 neurospheres per condition were collected and placed on poly-L-lysine coated coverslips in 24-well culture dishes (Millicell^®^, Merck Millipore, Burlington, MA) containing Neurobasal medium supplemented with 5% of fetal bovine serum (Gibco™, Thermo Fisher Scientific Waltham). The cultures were further maintained for 10 days, supplying 30% of total medium volume every 3 days and monitoring by phase-contrast microscopy. Finally, the coverslips containing differentiating cells were fixed with 4% paraformaldehyde and processed for immunofluorescence analysis. The samples were studied under a Leica TCS-SP8 confocal microscope using the acquisition software Leica Application Suite X Life Science (Leica Microsystems, Wetzlar, DE). Three different Z-stack images were obtained in each of the coverslips and were used to quantify the total number of βIII-tubulin + cells, GFAP + cells, and DAPI + nuclei (1:1,000; Dako Corp., Corston, UK). The findings were expressed as a ratio (%) between βIII-tubulin + or GFAP + cells normalized by the total number of cells (nuclei) per field.

### 2.11 Mouse embryonic fibroblast (MEF) culture and *in vitro* assay for cell viability

To gain insights into the phosphorylation of AMPK in embryonic cells derived from hyh mice also expressing the α-SNAP hypomorphism, we used MEFs as previously described [7]. Briefly, MEFs were isolated from E13.5 embryos and then cultured in DMEM Dulbecco`s Modified Eagle Medium, supplemented with 10% fetal bovine serum (FBS), 1% non-essential amino acids, and 100 μg/mL penicillin/streptomycin. Briefly, pregnant females were euthanized, embryos were isolated, and their organs were excised. The tissue was disaggregated mechanically in 100 mm culture dishes containing 2 mL of trypsin, later were transferred to 15-mL tubes and centrifuged for 5 min at 1,000 g. Viable cells were counted as previously described and seeded at a density of 50,000 cells/mL. All the experiment was done when the cells reached a confluence of 80%. Third passage MEFs were cultured in 24-well culture dishes at a density of 5,000 cells/well with DMEM supplemented, at 37°C for 24 h, in presence and absence of increasing doses of AICAR (1–500 mM) and Compound C (1–500 μM). Cell viability, as a response to the pharmacological treatment, was analyzed by the colorimetric 3-(4,5-dimethylthiazol-2-yl)-2,5-diphenyltetrazolium bromide method (MTT; Sigma, St. Louis) as an indirect measurement of cell viability based on cell mitochondrial activity. After treatment, cells were lysed and the absorbance was measured at 570 nm with a spectrophotometer (Biomate, Thermo Scientific, Waltham, US). Each sample was measured by triplicate and plotted as cell viability percentage resulting from the absorbance units of treated cells (T) normalized by the absorbance units of its controls (C), multiplied by 100 ([T/C] × 100), as was previously described by Concha et al. (Concha et al., 2018).

### 2.12 Pharmacological modulation of AMPK activity (in proliferation and differentiation assays)

Compound C (AMPK inhibitor) and AICAR (AMPK activator) (Calbiochem, Merck) were added to culture dishes in a concentration of 1–500 μM and 1–500 mM, respectively, for 24 h to MEF cultures. Cell viability was evaluated using MTT assay, and the effectiveness of the AMPK modulation was verified by Western blot. A dose-response curve was then generated. In neurosphere proliferation and differentiation assays, Compound C was used at 10 μM and AICAR at 5 mM. In proliferation assays, they were added to culture dishes after 6 days (co-incubation with BrdU). In neurosphere differentiation assays, they were added to culture dishes after 9 days of differentiation protocol. After 24 h, proliferating or differentiating neurospheres were fixed, immunostained, and visualized as previously described. Differentiating neurons (βIII-tubulin^+^ cells) and astrocytes (GFAP + cells) were counted within a radius of 120 μm from attached neurospheres. According to the migration rate of differentiating cells proposed by Galindo et al., this area includes only those cells generated during the last 24 h (Galindo et al., 2018).

### 2.13 Statistical analysis

Data were analyzed using Student’s t-test or one-way ANOVA with Bonferroni’s test using the GraphPad Prism software (CA, United States). All the data is presented as mean ± SEM. Differences with a *p*-value <0.05 were considered statistically significant.

## 3 Results

### 3.1 Expression of α-SNAP and consequences of M105I (hyh) mutation in the developing brain

**FIGURE 1:**
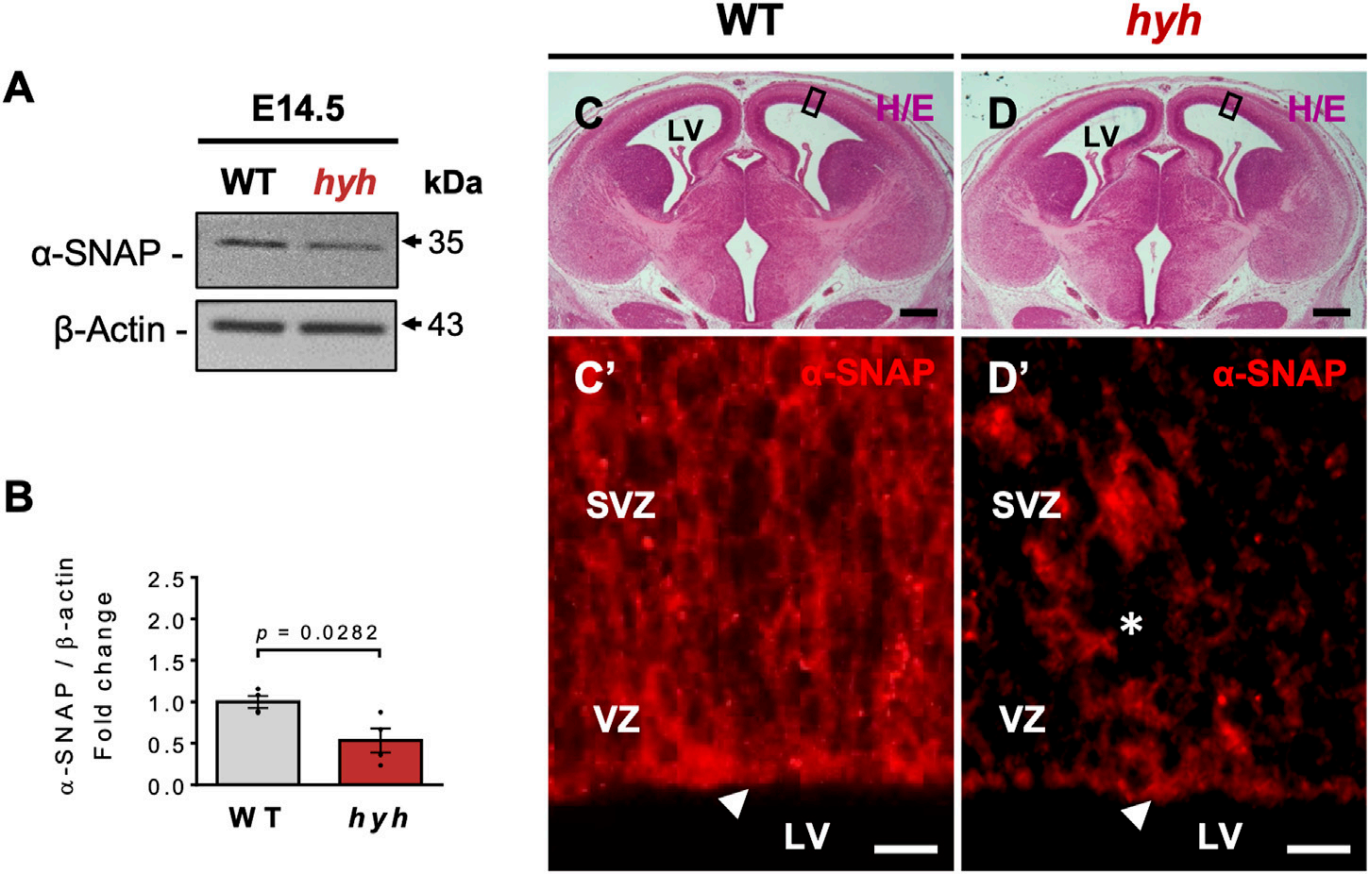
α-SNAP expression pattern and consequences of hyh (M105I) mutation. **(A)**. Western blot analysis of WT and hyh telencephalic lysates at E14.5 using antibodies against α-SNAP. β-actin was used as loading control. **(B)**. Densitometric analysis of Western blot in **(A)**. The data is presented as mean ± SEM, n = 4. Differences with *p* < 0.05 were considered significant (Student’s t-test with Welch’s correction). **(C,D)**. Representative brain coronal sections stained with hematoxylin/eosin (H/E) from WT **(C)** and mutant mice **(D)** at E14.5. The black boxes indicate the area displayed with higher magnification in **(C’)** and **(D’)**. **(C’,D’)**. Immunostaining for α-SNAP in the ventricular (VZ) and subventricular zone (SVZ). Note that hyh mice showed a reduction in α-SNAP immunoreactivity in the apical region of the VZ (white arrowheads) and the SVZ (asterisk). LV: Lateral Ventricles. Scale Bars: D and E = 300 µm; D’ and E’ = 10 µm.

α-SNAP is ubiquitously expressed in diverse cell types and organs (Arcos et al., 2017). Western blot analyses of different tissues/organs of newborn mice showed that α-SNAP was preferentially expressed in the brain (Supplementary Figure S1A), showing variations in the levels of α-SNAP protein in different brain regions and developmental stages (Supplementary Figures S1B, C). Since the pallium (dorsal region of developing telencephalon) of mice reaches the neurogenic peak at E14.5 (Gotz and Huttner, 2005), we evaluated the expression levels of α-SNAP at this stage in both WT and hyh telencephalon samples. Our results showed that the telencephalon of E14.5 hyh mice expressed a clear hypomorphism of α-SNAP, exhibiting ∼46% less amount of protein when compared with WT samples (Figures 1A, B). Immunofluorescence approaches showed that α-SNAP was preferentially expressed in the NSPCs residing in the apical region of the dorsal telencephalon VZ in E14.5 WT embryos and its immunoreactive pattern dramatically decreased in hyh littermates (Figures 1C–D’). Remarkably, other hyh mutant tissues also showed a reduction in α-SNAP levels (Supplementary Figure S1D), indicating that α-SNAP hypomorphism is not exclusive to the hyh brain. These findings suggest that α-SNAP is ubiquitous and widely distributed in the developing brain. The naturally occurring M105I (hyh) mutation provokes a reduction of α-SNAP levels in the brain and other tissues, affecting apical NSPCs.

**FIGURE 2:**
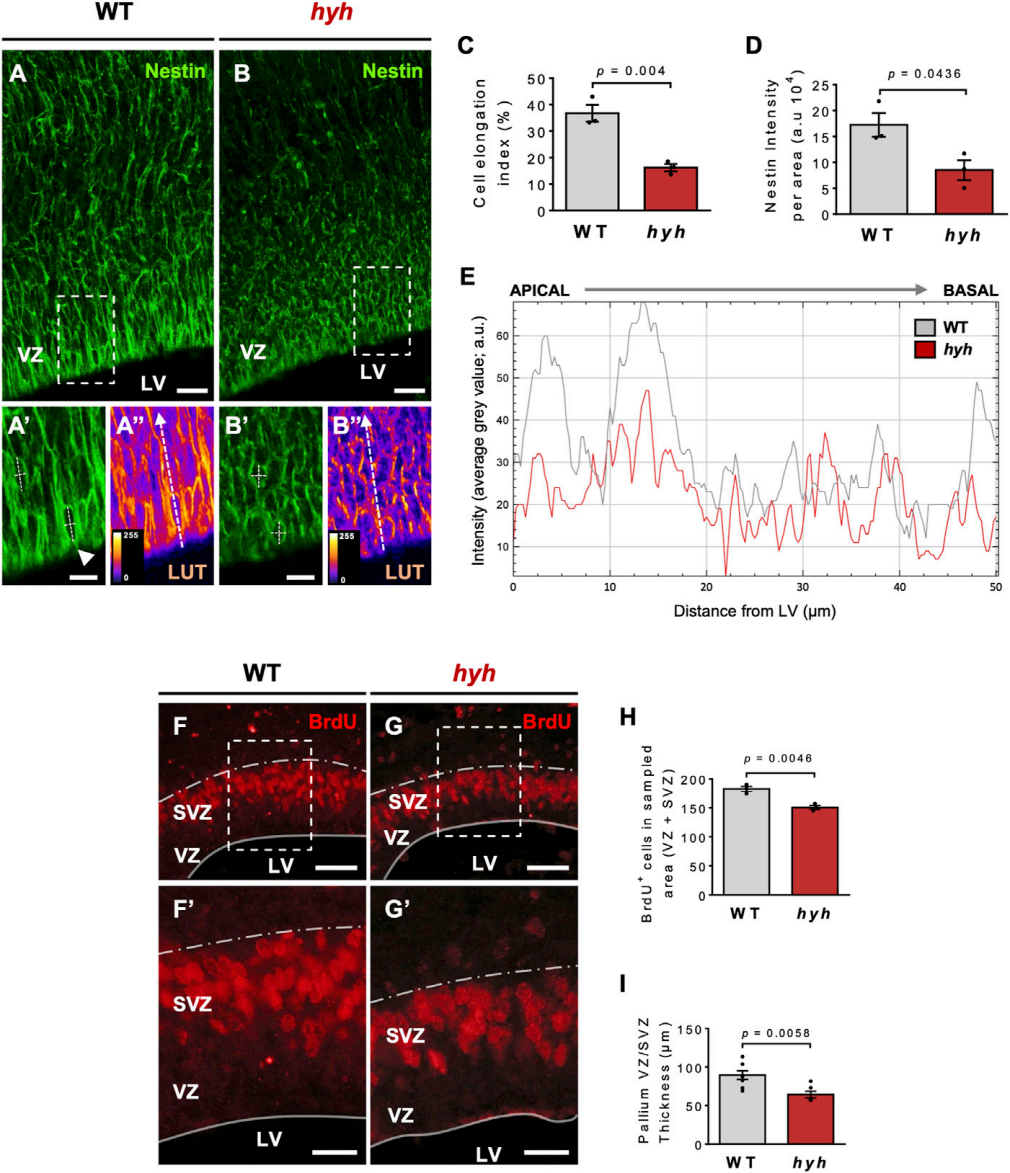
Consequences of hyh (M105I) mutation on apical NSPCs and ventricular zone (VZ) cytoarchitecture. (**A-B’’)**. Representative telencephalic coronal sections of WT and hyh mutant mice at E14.5 immunostained for Nestin, a marker of neural stem/progenitor cells (NSPCs). Dashed rectangles depict the zoomed area displayed in **(A’-A’’)** and **(B’-B’’)** (WT and hyh*,* respectively). **(A’’,B’’)** are the pseudocolored LUT (Look Up Table) versions of **(A’,B’)**, respectively, to highlight the intensity of the immunoreaction and the shape of VZ cells. Apical-basal and lateral axes of the Soma of single NSPCs in the VZ were measured as depicted in **(A’,B’)** (white dashed lines) to calculate cell elongation index (see C). The white dashed arrows from apical to basal in **(A’’,B’’)** indicate the regions used for plotting the immunofluorescence intensity profile shown in E. **(C)**. Cell elongation index (%) was calculated as EI=(AB–L)/(AB + L), where AB and L represent the apical-basal (major) and lateral (minor) axes of the ellipse-shaped Soma of individual NSPCs (see A’’ and B’’). Five to 10 cells per picture (40X), four pictures per animal, and three animals per condition (WT and hyh) were analyzed. **(D)**. Quantification of Nestin immunofluorescence intensity. One region of interest (ROI) of 14,000 μm^2^ (80 µm length from ventricular surface, 175 µm width) was considered in each section (4 sections per animal, three animals per condition). **(E)**. Plot of Nestin immunofluorescence intensity profile in the VZ (as depicted with dashed white arrows in A’’ and B’’ for WT and hyh, respectively). A distance of 50 µm from ventricular surface (apical to basal) was measured. Note the reduction of Nestin immunofluorescence intensity particularly in the most apical region. **(F,G)**. Representative coronal sections of WT **(F)** and hyh mutants **(G)** at E14.5 immunostained for BrdU. Dashed rectangles indicate the areas displayed with higher magnification in F**’** and G’. **(F’,G’)**. Higher magnifications of the VZ/SVZ at the level of the dashed rectangles in F and G. The thickness of the VZ/SVZ is depicted. **(H)**. Quantification of BrdU positive cells of VZ/SVZ normalized by sampled area in WT (n = 3) and hyh (n = 3) mice. **(I)**. Morphometric analysis of VZ/SVZ thickness in WT (n = 8) and hyh (n = 6) mice. Data is shown as mean +/- S.E.M. Differences with *p* < 0.05 were considered significant (Student’s t-test with Welch’s correction). LV, Lateral Ventricles, VZ, Ventricular Zone, SVZ Subventricular zone. Scale Bars: A-B = 20 µm; A’-B’ = 10 µm; E-F = 50 µm; E’-F’ = 20 µm.

**FIGURE 3:**
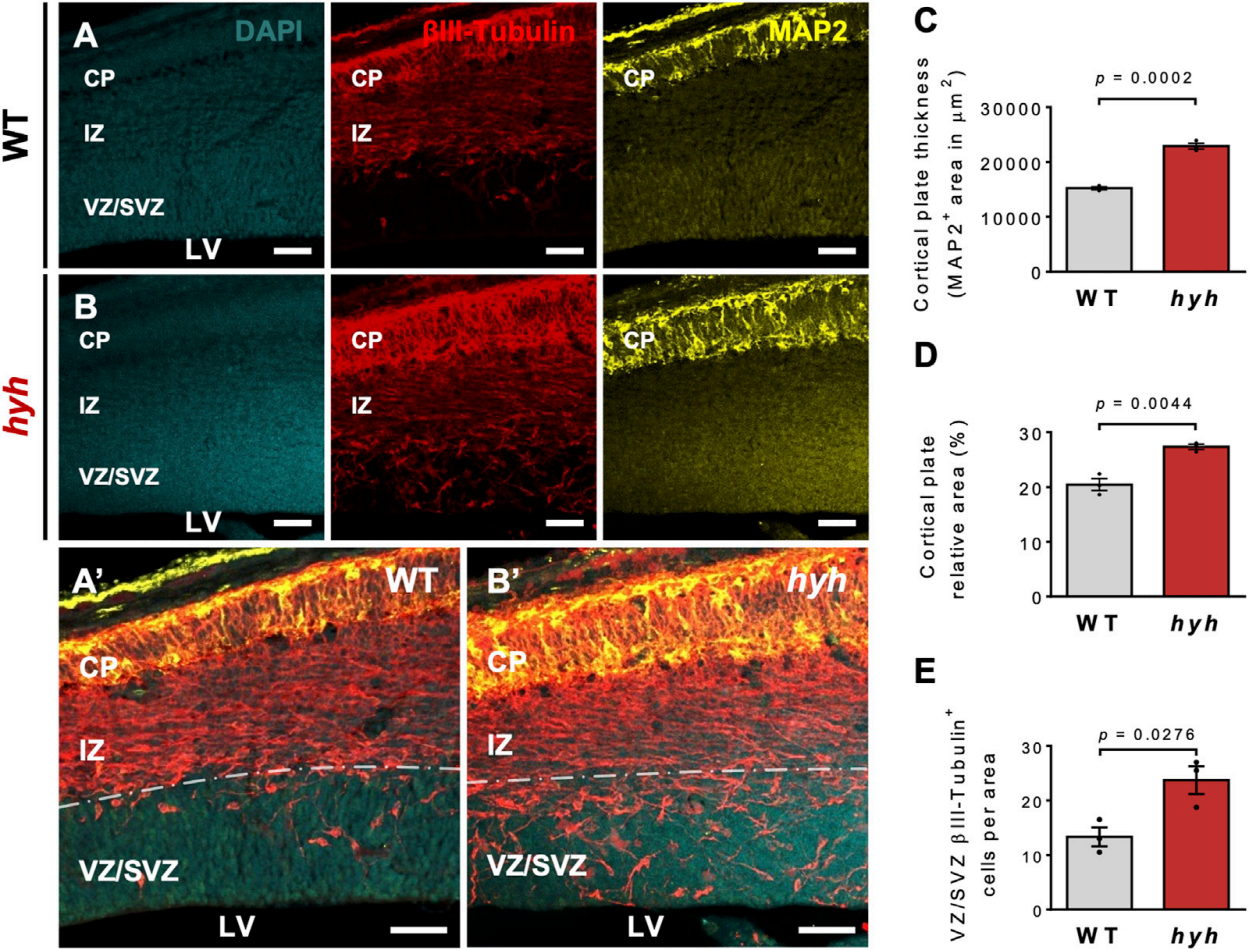
Increased neuronal differentiation in hyh embryonic brains. **(A,B)**. Representative coronal sections of WT **(A)** and hyh mutants **(B)** at E14.5 immunostained for βIII-tubulin (red) and MAP2 (yellow). Cell nuclei were stained with DAPI (blue). βIII-tubulin + cells are committed with the neuronal lineage and MAP2+ cells are maturing neurons. Ventricular zone/Subventricular zone (VZ/SVZ), intermediate zone (IZ), and cortical plate (CP) are depicted. Note that maturing neurons are mainly located in the CP. **(A’,B’)**. Higher magnifications of the merged images showed in A and B, respectively. Dashed line indicates the limit between SVZ and IZ. **(C)**. Morphometric analysis of CP (MAP2+ cells) thickness in WT (n = 3) and hyh (n = 3) mice. **(D)**. Morphometric analysis of CP relative area (CP area/pallium area) in WT (n = 3) and hyh (n = 3) mice. **(E)**. Quantification of βIII-tubulin + cells in the VZ/SVZ normalized by sampled area in WT (n = 3) and hyh (n = 3) mice. Data is shown as mean +/- S.E.M. Differences with *p* < 0.05 were considered significant (Student’s t-test with Welch’s correction). LV: Lateral Ventricles. Scale Bars: A-B = 50 µm; A’-B’ = 50 µm.

Previous work performed by our group and others suggests that the M105I mutation of α-SNAP affects the biology of apical NSPCs and their neurogenic process (Chae et al., 2004; Ferland et al., 2009); however, it is not clear whether these alterations are cell-autonomous or secondary to the pathological brain environment of hyh mutant mice. Thus, we aimed to characterize NSPCs and neurogenesis both *in vivo* and *in vitro* to exclude the influence of extrinsic environmental factors present *in vivo*. Immunofluorescence studies at E14.5, using Nestin as a marker of NSPCs, showed that the characteristic radial organization of apical NSPCs was altered in hyh embryos (Figures 2A, B). In fact, the characteristic elongated ellipse-shaped Soma of polarized WT-NSPCs was less pronounced in hyh-NSPCs, showing a more rounded (balloon-like) morphology (Figures 2A’–2B’). These changes are a proxy of cell apical-basal polarity and were quantified by (i) measuring apical-basal and lateral axes of the Soma of single NSPCs in the VZ, and (ii) calculating the elongation index (EI), described in Methods. The EI of NSPCs was reduced from a mean of 36.7% in WT-NSPCs to 16.2% in hyh-NSPCs (Figure 2C). Furthermore, these cells showed a dramatic decrease in Nestin immunoreaction (Figures 2A’’, B’’, D), particularly in the most apical region of the VZ/SVZ (Figure 2E), suggesting a premature differentiation of apical NSPCs. Interestingly, Western blot analyses of telencephalic samples at E14.5 also showed a dramatic reduction in Nestin expression in hyh mice (Supplementary Figures S2A, B). On the other hand, even though no statistical differences were observed in the number of mitotic figures in different regions of the VZ at E14.5 (Supplementary Figure S3A–D), experiments of BrdU incorporation after 2 h showed a reduction in the number of BrdU + cells in the VZ/SVZ of hyh mutant mice (Figures 2F–H). In addition, morphometric analyses revealed that the thickness of the VZ/SVZ was significantly reduced when compared with WT controls (Figures 2F’–G’, I). Western blot analyses showed no statistical differences in βIII-tubulin levels in telencephalic samples (Supplementary Figures S2A–C); however, we found that the thickness and the relative area of the developing cortical plate (βIII-tubulin+/MAP2+ cells) were increased in E14.5 hyh mice (Figures 3A–D). Furthermore, the number of βIII-tubulin + cells in the VZ/SVZ of hyh mice was also increased (Figures 3A’–B’, E), suggesting a premature commitment of NSPCs to the neuronal lineage.

**FIGURE 4:**
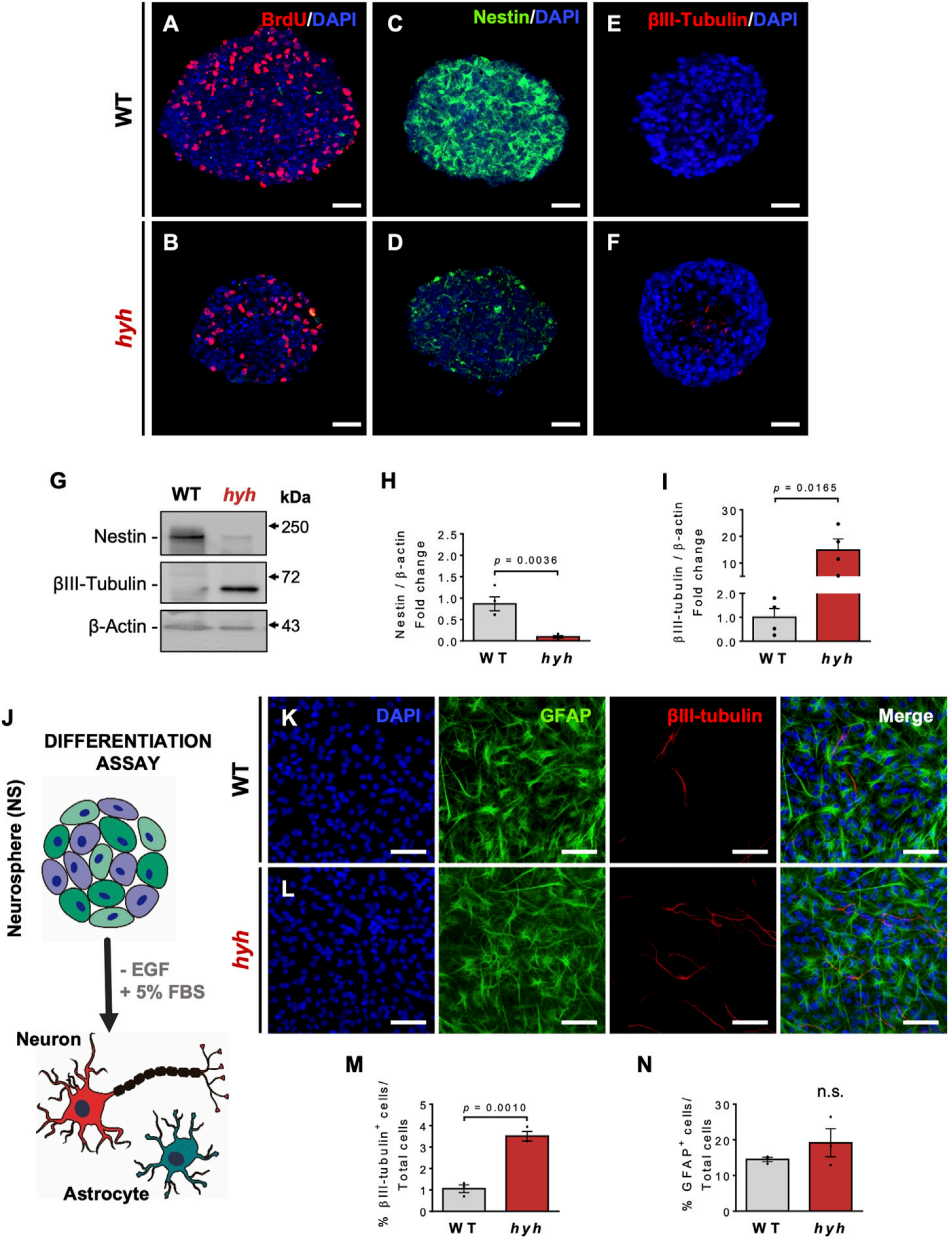
Increased neuronal differentiation of hyh-NSPCs is a cell-autonomous process. **(A–F)**. Representative immunostaining for BrdU **(A,B)**, Nestin **(C,D)** and βIII-tubulin **(E,F)** of NSPCs from WT and hyh mice cultured for 7 days *in vitro* (DIV) using the neurosphere (NS) assay. **(G)**. Western blot analysis of WT and hyh NS lysates using antibodies against Nestin and βIII-tubulin. β-Actin was used as loading control. **(H,I)**. Densitometric analysis of Western blots in **(G)**. The data is presented as mean ± SEM, n = 4. Differences with *p* < 0.05 were considered significant (Student’s t-test with Welch’s correction). **(J)**. Scheme depicting the differentiation assay of NS using a culture medium supplemented with 5% of fetal bovine serum (+FBS) and without epidermal growth factor (-EGF), for 10 days (10DIV). **(K,L)**. Immunofluorescence for GFAP and βIII-tubulin in WT and hyh NS after 10 days of differentiation. Cell nuclei were stained with DAPI. **(M,N)**. Quantification of GFAP- and βIII-tubulin-positive cells. The data is presented as the percentage of positive cells in the total number of cells (nuclei) (n = 3). Differences with *p* < 0.05 were considered significant (Student’s t-test with Welch’s correction). n. s. = no significant differences. Scale Bars: A-F = 30 µm; K-L = 50 µm.

To analyze whether the consequences of the M105I mutation in the neurogenic capacity of hyh-derived NSPCs (hyh-NSPCs) were cell autonomous, we used neurosphere culture systems as previously described (Henzi et al., 2018). We found that after 7 days *in vitro* (DIV), hyh-NSPCs exhibited reduced proliferative rate, as measured by BrdU incorporation (Figures 4A, B and Figures 7A, B, E), reduced Nestin immunoreactivity (Figures 4C, D), and increased immunoreaction to βIII-tubulin compared with controls (Figures 4E, F). These results were confirmed by Western blot analyses (Figures 4G–I). Next, we evaluated the capacity of neurospheres to generate neurons under serum-enriched conditions, a well-known stimulus for inducing differentiation (Pirhajati Mahabadi et al., 2015) (Figure 4J). Consistently, after 10 days in culture (10 DIV), hyh-NSPCs generate more neurons (βIII-tubulin + cells) than controls (WT-NSPCs) (Figures 4K–M), while no significant differences were observed in the generation of astrocytes (GFAP + cells) (Figures 4K–L, N).

### 3.2 The M105I mutation of α-SNAP leads to AMPK overactivation in NSPCs

**FIGURE 5:**
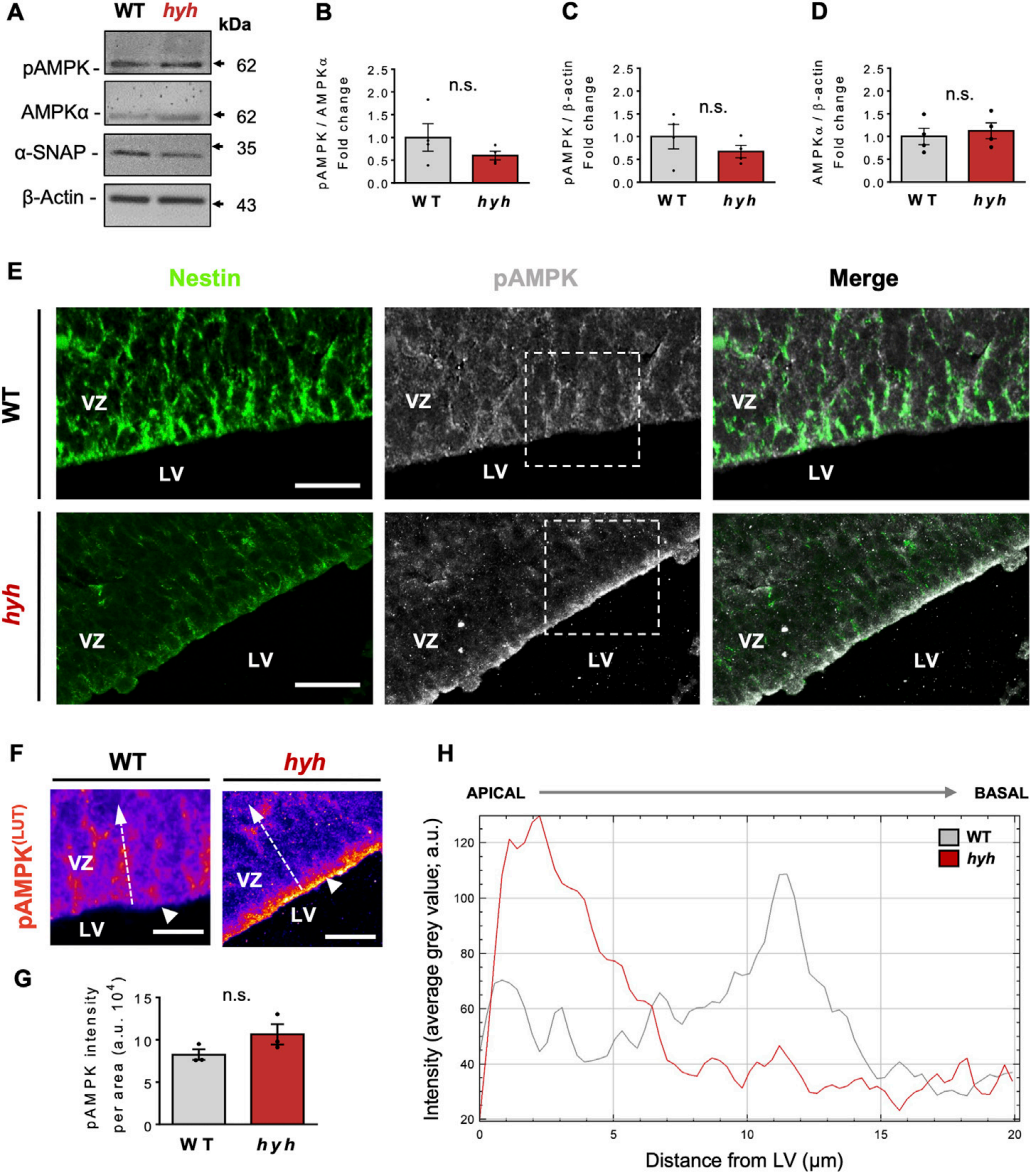
Expression and distribution pattern of phosphorylated AMPK in the telencephalon of hyh mice. **(A)**. Western blot analysis of proteins obtained from WT and hyh telencephalon at E14.5 using antibodies against pAMPK, total AMPKα, and α-SNAP. β-actin was used as loading control. **(B–D)**. Quantification of pAMPK protein levels normalized on total AMPK **(B)** and β-actin levels **(C)**, and total AMPK on β-actin protein levels **(D)**. The bars represent the mean ± SEM (n = 4). Differences with *p* < 0.05 were considered significant (Student’s t-test with Welch’s correction). n. s. = no significant differences. **(E)**. Dorsal ventricular zone (VZ) of brain coronal sections from WT **(E)** and hyh **(F)** mice at E14.5 immunostained for Nestin (green) and pAMPK (grey). Dashed rectangles depict the zoomed area displayed in F (WT and hyh*,* respectively). **(F)**. Pseudocolored LUT (Look Up Table) high magnification images of the areas indicated by dashed rectangles in E (pAMPK). Note the changes in pAMPK immunoreactive pattern in the most apical region of the VZ in hyh mice (white arrowheads). **(G)**. Quantification of pAMPK immunofluorescence intensity in the VZ. One region of interest (ROI) of 2,000 μm^2^ (20 µm length from ventricular surface, 100 µm width) was considered in each section (4 sections per animal, three animals per condition). The bars represent the mean ± SEM (n = 3). Differences with *p* < 0.05 were considered significant (Student’s t-test with Welch’s correction). n. s. = no significant differences. **(H)**. Plot of pAMPK immunofluorescence intensity profile in the apical VZ (as depicted with dashed white arrows in F for WT and hyh, respectively. A distance of 20 µm perpendicular to the ventricular surface (apical to basal) was measured. Note the increased immunofluorescence intensity of pAMPK in the most apical region of the VZ in hyh mice. LV, Lateral ventricle. Scale Bars: E = 20 µm, F = 10 µm.

**FIGURE 6:**
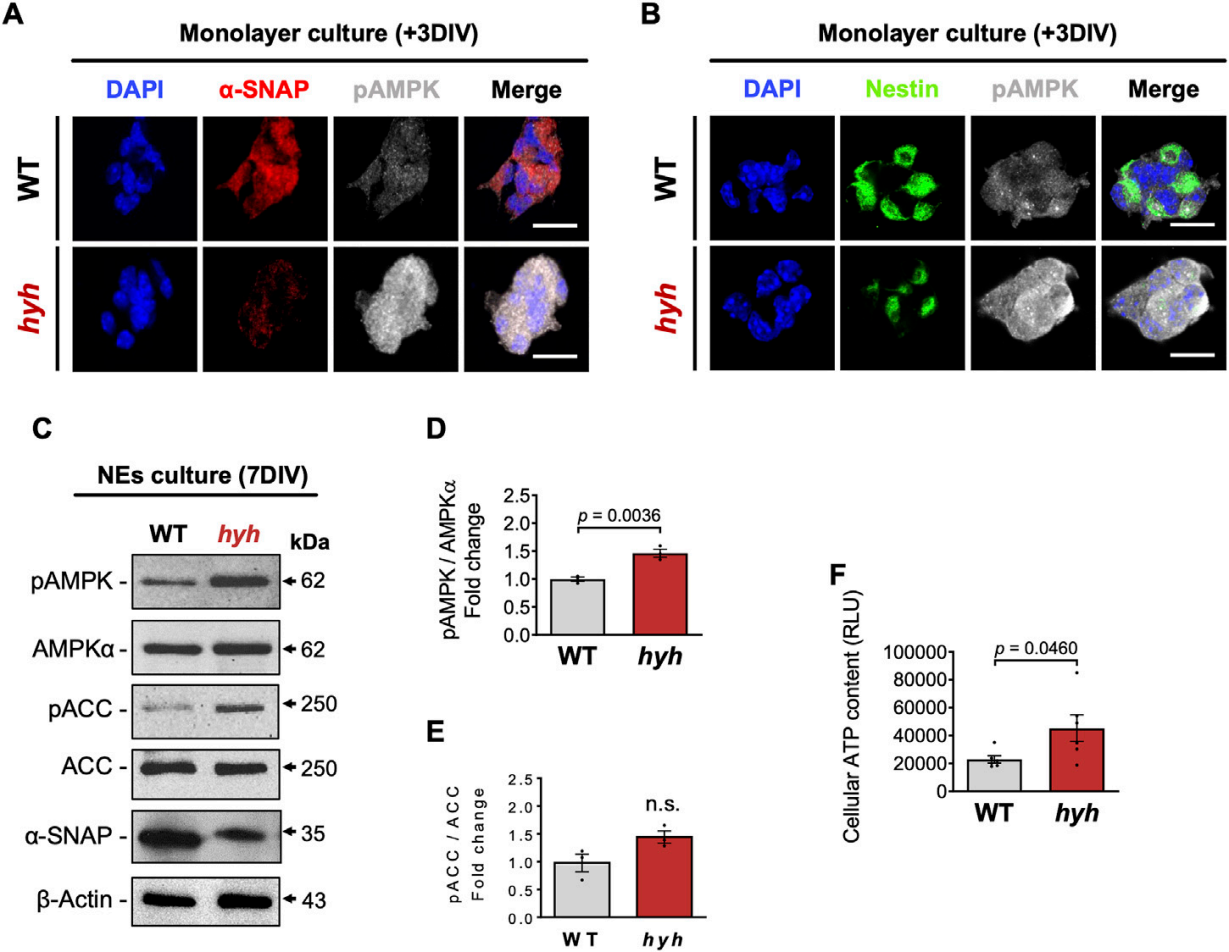
AMPK overactivation in NSPCs derived from hyh mice. **(A,B)**. Neurospheres (7DIV) were disaggregated and cultured as monolayer for 3DIV. Representative double immunofluorescence stainings of pAMPK combined with α-SNAP **(A)** and Nestin **(B)**. Scale Bars: D-E = 20 µm. **(C)**. Western blot analysis of seven DIV neurospheres obtained from WT and hyh mice using antibodies against α-SNAP, pAMPK, AMPKα, pACC and ACC. **(D,E)**. Densitometric analysis of pAMPK **(D)** and pACC **(E)** protein levels normalized on AMPK and ACC total protein levels, respectively. **(F)**. Measurement of cellular ATP content of seven DIV neurospheres. RLU: relative luminescence units. The bars represent mean ± SEM (n = 3 in D-E; n = 6 in F). Differences with *p* < 0.05 were considered significant (Student’s t-test with Welch’s correction). n. s. = no significant differences.

α-SNAP has been identified as a negative regulator of AMPK activity, by directly interacting with pAMPK and having phosphatase activity (23). In addition, it has also been suggested that the M105I mutation of α-SNAP increases its phosphatase activity (23); nevertheless, AMPK activity in hyh-NSPCs is unknown. On the other hand, several studies indicate that AMPK activity regulates the proliferation/differentiation balance of NSPCs (Zang et al., 2008; Zang et al., 2009; Liang et al., 2018; Shi et al., 2019; Wang et al., 2019). Immunoblot analyses of E14.5 telencephalic homogenates showed no significant differences in the amount of AMPKα and p-AMPKα between hyh and WT mice (Figures 5A–D). However, a detailed analysis of the immunoreactive pattern of pAMPK in the Nestin + NSPCs of the VZ (Figures 5E, F) showed that even though pAMPK immunofluorescence intensity in the VZ did not show significant differences between WT and hyh mice (Figures 5F, G), pAMPK intensity was increased in the most apical region of VZ in hyh mice (Figures 5E, F, H). To test whether AMPK activity was increased in hyh-NSPCs, we isolated NSPCs directly from the dorsal VZ and performed monolayer and neurospheres culture systems. Interestingly, immunofluorescence analyses in NSPCs monolayer cultures showed reduced levels of α-SNAP (Figure 6A) and Nestin (Figure 6B), and increased levels of pAMPK immunoreaction (Figures 6A, B).

In addition, AMPK phosphorylation was also analysed by Western blot in neurospheres. As expected, the levels of α-SNAP (M105I) were reduced in hyh neurospheres (Figure 6C). Like the results obtained in monolayer cultures, neurospheres from hyh mice showed increased levels of p-AMPKα when compared with WT neurospheres (Figures 6C, D). Phospho-ACC, a downstream target of p-AMPK, showed a 1.5-fold change in hyh-NSPCs, but the difference was not statistically significant (Figures 6C–E). Since AMPK activation switches off anabolic pathways that consume ATP and switches on catabolic pathways that generate ATP (Hardie, 2003), we measured the cellular ATP content in order to test the bioenergetic impact of p-AMPK up-modulation in hyh-NSPCs. Remarkably, we found that hyh-NSPCs showed increased levels of ATP compared to WT-NSPCs, as measured by a luminescence assay (Figure 6F). These results strongly suggest that AMPK overactivation in hyh-NSPCs (i) modifies the cellular bioenergetic status, and (ii) is not occurring in response to low ATP levels. To test whether the overactivation of AMPK was associated with or secondary to the activation of other kinases, we evaluated the phosphorylation status of AKT and ERK1/2. These serine/threonine protein kinases are involved in the regulation of the proliferation/differentiation balance of NSPCs (Wang et al., 2009; Ojeda et al., 2011) and can also modulate AMPK activity (Kawashima et al., 2015; Zhao et al., 2017). Remarkably, the levels of pAKT and pERK1/2 were similar in hyh and WT NSPCs (Supplementary Figure S4), suggesting that AMPK overactivation is not related with overactivation of other kinases.

### 3.3 Pharmacological modulation of AMPK rescues the overproduction of neurons in hyh-NSPCs

The abovementioned results indicate that hyh-NSPCs have increased AMPK activity and premature neuronal differentiation. Therefore, we decided to investigate whether the overproduction of neurons in hyh-NSPCs could be attenuated or reverted by an AMPK inhibitor (Compound C). On the other hand, we also tested whether AMPK activation by 5-aminoimidazole-4-carboxamide-1-β-D-ribofuranoside (AICAR) in WT-NSPCs could resemble (phenocopy) the phenotype of hyh-NSPCs, increasing neuronal differentiation.

To determine the impact of Compound C and AICAR on cell viability and their efficiency for modulating AMPK activity, we first established dose-response curves for both drugs in WT mouse embryonic fibroblasts (MEFs) (Supplementary Figure S5). The 50% inhibitory concentration (IC50) values in MTT assays (that is, the concentration of drug which reduce cell viability to 50%) were 9.0 mM for AICAR and 43.5 μM for Compound C. Thus, we tested AMPK activity by using doses below IC50 values and quantifying the phosphorylation of AMPKα (Thr172) and downstream targets such as p-ACC (Ser79), and p-TSC2 (Ser1387). We found that 10 μM Compound C induced a decrease in p-AMPK (Thr172), p-ACC (Ser79), and p-TSC2 (Ser1387) levels (Supplementary Figure S6). On the other hand, 5 mM AICAR induced an increase in p-AMPK (Thr172), p-ACC (Ser79), and p-TSC2 (Ser1387) levels (Supplementary Figure S7).

**FIGURE 7:**
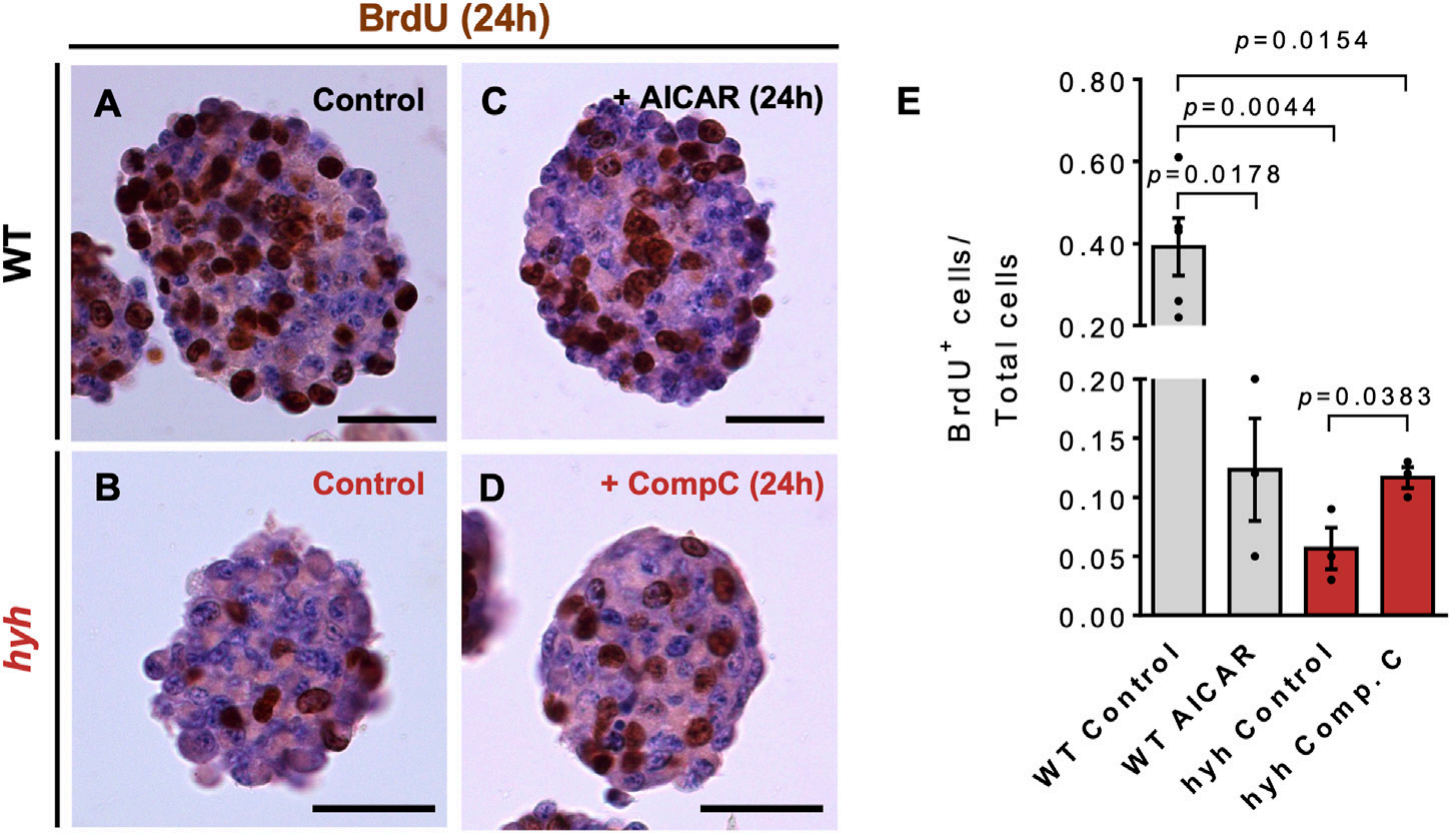
NSPCs derived from hyh mice show reduced proliferation rate, which is reversed through pharmacological modulation of AMPK. **(A–D)**. Representative sections of neurospheres WT **(A,C)** and hyh **(B,D)** mice cultured for 7 days *in vitro* (DIV) and immunostained for BrdU and counterstained with Hematoxylin. BrdU (20 mmol/L) was added to the cultures 24 h before fixation. A and B are representative sections from WT **(A)** and hyh **(B)** neurospheres with no treatment (control). C and D are representative images of WT neurospheres treated for 24 h with 5 mM AICAR **(C)** and hyh neurospheres treated with 10 μM Compound C **(D)**. **(E)**. Quantification of BrdU labeling index (i.e., the fraction of BrdU-positive cells over the total number of cells). The bars represent mean ± SEM (n = 3–5). Differences with *p* < 0.05 were considered significant (One-way ANOVA with Bonferroni’s post-test). Scale Bars: A-D = 30 µm.

**FIGURE 8:**
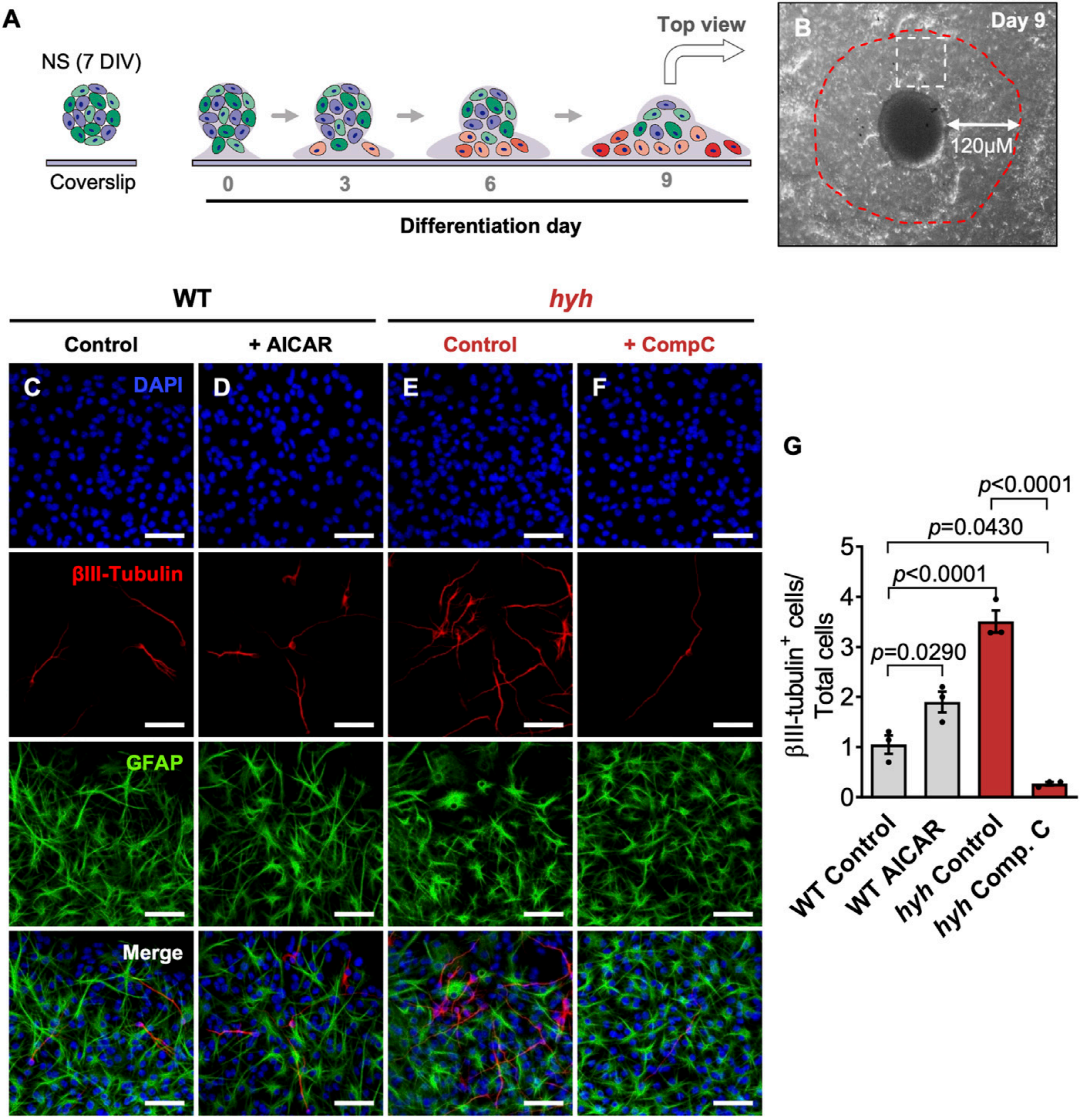
NSPCs derived from hyh mice differentiate preferentially towards the neuronal lineage, which is reversed through pharmacological modulation of AMPK. **(A)**. Scheme depicting the experimental design. Neurospheres (NS) (7DIV) from WT and hyh mice were transferred onto poly-l-lysine-coated coverslips and cultured for 10 days (day 0 to day 9) in the presence of 5% of bovine fetal serum to induce differentiation. After 10 days under differentiation treatment, WT NS were treated with 5 mM AICAR and hyh NS with 10 μM Compound C, for 24 h. Vehicle was used as control. **(B)**. Representative phase contrast image of 10DIV differentiating NS (top view). Differentiating neurons (βIII-tubulin + cells) were counted within a radius of 120 μm from neurospheres (red dashed line). White square represents an area similar to those shown in C-F. **(C–F)**. Representative immunostaining for βIII-Tubulin and GFAP of differentiated WT and hyh NS under pharmacological modulation of AMPK. DAPI was used to stain nuclei. **(G)**. Quantification of βIII-tubulin + cells. The bars represent the percentage of βIII-tubulin + cells over the total number of cells (nuclei) (n = 3 for each condition). Differences with *p* < 0.05 were considered significant (One-way ANOVA with Bonferroni’s post-test). Scale Bars: C-F = 50 µm.

Then, we performed proliferation and differentiation analyses in neurospheres previously treated with 10 μM Compound C (hyh-NSPCs) or 5 mM AICAR (WT-NSPCs) for 24 h (Figures 7, 8). To evaluate the proliferative capacity of WT and hyh-NSPCs, we applied a pulse of BrdU (20 mmol/L) at 6 DIV neurospheres and quantified the number of cycling cells (BrdU+) after 24 h. As stated before, hyh-NSPCs showed a dramatic reduction in the number of BrdU + cells compared with WT-NSPCs (Figures 7A, B, E); however, when treated with Compound C, the number of BrdU+ was increased 2.1 times (Figures 7B, D, E). On the other hand, when WT NSPCs were treated with AICAR the number of BrdU + cells was reduced from 39% to 12% (Figures 7A, C, E).

For differentiation analyses, neurospheres of seven DIV were incubated under non-proliferative conditions. After 10 DIV, differentiating neurospheres were fixed and neurons (βIII-tubulin^+^ cells) and astrocytes (GFAP + cells) were counted within a radius of 120 μm from neurosphere surface (Figures 8A, B). According to the migration rate of differentiating cells proposed by Galindo et al., this area includes only those cells generated during the last 24 h (Galindo et al., 2018). Interestingly, no differences were observed with pharmacological treatments in the number of astrocytes (GFAP + cells) (data not shown); however, reduction of AMPK activity in hyh-NSPCs (*via* Compound C) abolished the overproduction of neurons (βIII-tubulin + cells) (Figures 8E–G). By contrast, AMPK activation in WT-NSPCs (*via* AICAR) enhanced their neurogenic capacity as evidenced by an increase in the generation of βIII-tubulin + cells compared to non-treated controls (Figures 8C, D, G). Together, these data strongly suggest that the M105I mutation of α-SNAP provokes a premature neuronal commitment and reduces the proliferative rate of NSPCs by, at least in part, inducing an overactivation of pAMPK.

## 4 Discussion

### 4.1 NSPCs from hyh mice exhibit a preferential commitment with the neuronal lineage

The capacity of neural stem-progenitor cells (NSPCs) to undergo self-renew or commit to a specified cell fate is essential for maintaining NSPCs and neurogenesis throughout development. A delicate interplay of intrinsic and extrinsic cues controls the fate transition of NSPCs during embryonic neurogenesis (Gotz and Huttner, 2005); however, how these cues are integrated remains mostly unknown. The present study highlights the role of α-SNAP and AMPK in NSPCs biology and supports that overactivation of AMPK underlies, at least partially, a premature and preferential commitment of NSPCs to the neuronal lineage in α-SNAP (hyh) mutant embryos.

Previous research has suggested that α-SNAP protein is ubiquitously expressed in multiple cell types and organs (Nishiki et al., 2001; Batiz et al., 2009a; Arcos et al., 2017; de Paola et al., 2019). Our results indicate that even though α-SNAP is expressed in every tissue analyzed, it is mainly expressed in the brain. This fact is interesting since ơ-SNAP expression follows the pattern of generation of neurons in the developing central nervous system (Gotz et al., 1998; Hartfuss et al., 2001; Wagner et al., 2003; Chae et al., 2004; Gotz and Huttner, 2005; Ferland et al., 2009), suggesting that its expression is relevant for the neurogenic process. Furthermore, immunolocalization of α-SNAP showed that it is preferentially localized in the apical region of the ventricular zone, where resides apical NSPCs. Following previous reports, telencephalic samples of hyh mutant embryos showed a 50% reduction in α-SNAP protein levels, affecting the normal distribution of the protein in the apical domains of the ventricular zone. Therefore, pathological changes in the brain cortical development of hyh mutants, characterized by a premature overproduction of early-born neurons and disruption of the ventricular zone (Chae et al., 2004; Hong et al., 2004; Ferland et al., 2009), appear to be related to a reduction in the function (hypomorphism) of α-SNAP in NSPCs.

Altered cell fate of NSPCs in hyh embryos has been previously documented by other authors (Chae et al., 2004); however, the molecular mechanisms linking α-SNAP mutation with changes in cell fate are not fully understood. Chae et al. (2004) found that altered neural cell fate in hyh mice was accompanied by abnormal localization of several apical proteins in the VZ, but whether this phenomenon was cell-autonomous or secondary to extrinsic signals present in the pathological developing brain environment was not addressed (Chae et al., 2004). Here, we show that isolated hyh mutant NSPCs have reduced expression of Nestin (stem cell marker), reduced proliferative rate and increased expression of beta-tubulin-III (neuronal marker) after 7 days *in vitro*. *In vitro* differentiation assays show that NSPCs from hyh mice exhibit a preferential commitment with the neuronal lineage, producing more neurons than those NSPCs derived from wild-type mice. Interestingly, no differences were observed in the commitment with the glial lineage.

### 4.2 NSPCs from hyh mice show an overactivation of AMPK

Premature neuronal commitment appeared to be linked to a specific overactivation of AMPK. In fact, no differences were observed between wild type and hyh mice in the phosphorylation status of Erk and Akt, two kinases previously associated with the balance between self-renewal and neuronal differentiation of NSPCs (Rhim et al., 2016). Furthermore, Compound C suppressed the overgeneration of βIII-tubulin + cells and increased the proliferative rate in hyh-NSPCs cultures. At the same time, AICAR reduced BrdU incorporation and increased the neurogenic capacity of wild type cultures, supporting that the altered cell fate in hyh NSPCs is an AMPK-dependent cell-autonomous process and is not secondary to the abnormal developing brain environment of hyh embryos.

Adenosine monophosphate-activated protein kinase (AMPK) is a serine/threonine kinase protein complex activated by increased intracellular AMP/ATP (adenosine triphosphate) ratio that plays a central role in regulating cellular energy homeostasis (Hardie et al., 2012; Hardie, 2015). AMPK consists of three subunits (α, β and γ), with the α subunit displaying catalytic activity. Phosphorylation of Thr residue at amino acid 172 in the α subunit by upstream kinases (LKB1, CaMKKβ, TAK1) is essential for AMPK activation (Woods et al., 2000; Viollet et al., 2010). On the other hand, protein phosphatases have an important role in regulating AMPK activity by reducing phosphorylation at Thr-172. Protein phosphatases 2A (*p*P2A) and 2C can dephosphorylate AMPK in different cell types (Kudo et al., 1996; Wang and Unger, 2005; Ravnskjaer et al., 2006), but the exact mechanisms that modulate their action remain poorly understood.

Since AMPK functions as a hub to integrate cell signaling, metabolism, and transcriptional regulation, it is extraordinarily relevant in stem cells, matching metabolic plasticity with the energetic demands of stemness and lineage specification (Dasgupta and Milbrandt, 2009; Kim et al., 2012; Liang et al., 2018). For instance, the connection of the response to metabolic stress with signaling pathways that induce cell cycle arrest and differentiation is mediated by the activation of AMPK (Zang et al., 2009; Grigorash et al., 2018). On the other hand, cellular responses to AMPK activation are dependent on the degree and duration of AMPK activation. In this context, several *in vitro* and *in vivo* studies support that prolonged activation of AMPK can have detrimental impacts (Jung et al., 2004; Nakatsu et al., 2008; Amato and Man, 2011). Here we show that AMPK overactivation impacts the bioenergetic status of hyh-NSPCs. Remarkably, we found that increased levels of p-AMPK co-exist with increased cellular ATP content in hyh-NSPCs. Since increased ATP levels inhibit AMPK activity in several cell-types (Herzig and Shaw, 2018), these results strongly indicate that overactivation of AMPK in hyh-NSPCs is not a response to low ATP levels. On the other hand, since high ATP levels are not able to inhibit AMPK, a putative defect in the function of negative regulators of AMPK is highlighted.

A growing body of evidence supports that α-SNAP have several ‘non-canonical’ functions independent of NSF and SNAREs (Naydenov et al., 2012a; Naydenov et al., 2012b; Wang and Brautigan, 2013; Naydenov et al., 2014; Li et al., 2016). Wang and Brautigan reported that α-SNAP could directly inhibit AMPK signaling through dephosphorylation of the α subunit (AMPKαThr172) *in vitro*, suggesting that ơ-SNAP is a negative regulator of AMPK activity (Wang and Brautigan, 2013). Furthermore, by overexpression experiments in HEK293T cells, they suggested that the hyh (M105I) mutation of α-SNAP provokes a gain-of-function by increasing the binding to the AMPKα subunit and preventing AMPK activity more effectively than the wild type protein (Wang and Brautigan, 2013). However, as stated before, in hyh mice, the M105I mutation is associated with α-SNAP hypomorphism in most tissues, including the developing brain. Thus, we tested if α-SNAP M105I mutation leads to AMPK overinhibition (increased phosphatase activity of α-SNAP) or overactivation (α-SNAP hypomorphism) under *in vivo* natural conditions. Interestingly, immunoblot analyses did not show differences in pAMPK levels between wild-type and hyh embryonic telencephalon. However, imaging studies showed a strong immunoreaction for pAMPK in the apical region of the ventricular zone, suggesting increased activity of AMPK in apical NSPCs. This was further confirmed in cultured NSPCs, where we also found increased phosphorylation of AMPK and AMPK-downstream targets such as ACC, indicating that NSPCs from hyh mutant mice present an overactivation of AMPK. These findings diverge from those obtained by Wang and Brautigan (2013), suggesting that the M105I mutation confers to α-SNAP a gain of phosphatase function (Wang and Brautigan, 2013). However, they did not use natural α-SNAP M105I mutant cells (α-SNAP M105I hypomorphism) but cells overexpressing the mutant protein, thus generating a condition that may not represent α-SNAP function in hyh cells. We do not rule out whether the M105I mutation confers a gain of phosphatase function in NSPCs under *in vivo* conditions; however, our results suggest that the hypomorphism of α-SNAP induced by the M105I mutation overcomes a possible gain of phosphatase function, thus leading to overactivation of AMPK.

On the other hand, our results are apparently in disagreement with the results of a previous study in which is suggested that AMPK is not required for embryonic neurogenesis (Williams et al., 2011). These authors used an AMPKα1/α2-null mouse model and did not find alterations in cortical layer formation. However, they did not explore a gain-of-function approach of AMPK during development. Interestingly, although no changes were observed in cortical organization of AMPKα-null mice, these authors showed that AICAR affects neuronal maturation by inhibiting axogenesis and axon growth in an AMPK-dependent manner (Williams et al., 2011). Thus, even though a reduction in AMPK activity may not be reflected in an altered cell fate of NSPCs, it does not exclude that an overactivation of AMPK can have detrimental consequences such as premature neuronal differentiation, as shown by our results in hyh mice. In this context, several studies support a key role of AMPK in regulating the proliferation/differentiation balance of NSPCs. Pharmacological and genetic studies agree with our results and show that AMPK activation reduces proliferation of embryonic and adult NSPCs (Zang et al., 2008; Dasgupta and Milbrandt, 2009; Zang et al., 2009; Liang et al., 2018; Shi et al., 2019; Wang et al., 2019). Studies in postnatal animals have reported that activation of AMPK can increase the number of DCX + neurons in the hippocampus of young mice but not old mice (Jang et al., 2016; Odaira et al., 2019). On the other hand, Wang et al. have demonstrated that AMPK signaling plays a key role in regulating age-related changes in hippocampal neurogenesis. In this context, and similarly to what we observed in hyh embryos, inhibiting AMPK signaling by Compound C increase proliferative rate of aged hippocampal NSPCs (Wang et al., 2019). It is noteworthy that although NSPCs show many phenotypical differences throughout life, adult NSPCs are originated at early stages of brain development, allowing a certain degree of analogy with embryonic NSPCs (Merkle et al., 2004). On the other hand, AMPKβ1-deficient mice develop up to 25% fewer neurons in the cortex and up to 40% fewer in the cerebellum, dentate gyrus, and hypothalamus (Dasgupta and Milbrandt, 2009). Interestingly, these mice did not show consequences on the production of GFAP + glial cells (Dasgupta and Milbrandt, 2009).

### 4.3 Mechanisms linking α-SNAP-AMPK axis with neuronal differentiation

Several lines of evidence indicate that neuronal differentiation is a complex process that depends, among others, on the regulation and modulation of (i) apical-basal cell polarity, (ii) energy metabolism and mitochondrial function and dynamics, (iii) autophagy, and (iv) epigenetic changes in NSPCs. Remarkably, AMPK participates in all of them.

#### 4.3.1 Apical-basal polarity

The apical-basal polarity of NSPCs is essential for symmetric vs asymmetric cell divisions and, consequently, for regulating their proliferation/differentiation balance (Gotz and Huttner, 2005; Bustamante et al., 2019). The formation, maintenance and dynamic turnover of adherens junctions serve as intracellular anchoring sites for apical-basal polarity determinants to establish and maintain cell polarity (Bustamante et al., 2019; Hakanen et al., 2019). LKB1 and AMPK have been proposed as key regulators of apical-basal polarity in *Drosophila* oocyte and mammalian epithelial cells (Lee et al., 2007; Williams and Brenman, 2008; Nakano and Takashima, 2012). Interestingly, α-SNAP M105I mutation is associated with abnormal localization of many proteins implicated in regulation of apical-basal polarity of NSPCs (Chae et al., 2004) and abnormal localization of cortical granules in oocytes (de Paola et al., 2019); thus, highlighting a possible pathogenic role of AMPK overactivation in the hyh phenotype.

#### 4.3.2 Energy metabolism and mitochondrial biogenesis/function

Current evidence demonstrates that in addition apical-basal polarity and extracellular cues, metabolic pathways provide signals for stem cell renewal/differentiation balance. Thus, undifferentiated stem cells show relative low levels of ATP and a more glycolytic status. On the other hand, differentiated cells have increased mitochondrial oxidative phosphorylation and abundant ATP levels (Hu et al., 2016). This evidence indicates that mitochondria are central regulators of NSCs fate decisions in neurodevelopment and adult neurogenesis (Khacho et al., 2017). Increased mitochondrial number (increased biogenesis) and/or increased mitochondrial fragmentation (fission) have been associated with differentiation of NSCs. Khacho et al. demonstrated that changes in mitochondrial dynamics (increased fragmentation) of NSCs drive a reactive oxygen species (ROS)-mediated process, which suppresses self-renewal and promotes differentiation *via* NRF2-mediated retrograde signaling (Khacho et al., 2017). Interestingly, a growing body of evidence highlights the role of AMPK in regulating mitochondrial biogenesis and dynamics in several cell types. In fact, AMPK can phosphorylate and increase the activity of PGC1α, a master regulator of mitochondrial biogenesis (Jager et al., 2007). In mice, chronic activation of AMPK or overexpression of a constitutively active AMPKγ3 subunit leads to increased mitochondrial biogenesis in muscle cells (Bergeron et al., 2001; Garcia-Roves et al., 2008). A muscle-specific knockout of AMPKα subunits induces defects in mitochondrial biogenesis and function (Lantier et al., 2014). On the other hand, AMPK activation is sufficient to induce mitochondrial fission during energy stress (Toyama et al., 2016). These findings established AMPK as a crucial and direct regulator of mitochondrial biogenesis/dynamics. Interestingly, Wang and Brautigan (2013) demonstrated that the knockdown of α-SNAP enhances oxygen consumption and mitochondrial biogenesis in an LKB1/AMPK-dependent manner (Wang and Brautigan, 2013).

#### 4.3.3 Macroautophagy

There is also evidence for an active role for macroautophagy (autophagy) in regulating NSPC differentiation (Lv et al., 2014). The initial period of neuronal differentiation in mouse embryonic olfactory bulb is characterized by an increased expression of autophagy genes, and pharmacological inhibition of autophagy markedly decrease neuronal differentiation (Vazquez et al., 2012). AMPK is one of the critical regulators of autophagy in mammalian cells by controlling different steps. Activation of AMPK promotes autophagy by phosphorylating proteins of several autophagy-related complexes, such as mTORC1, ULK1, and PIK3C3/VPS34 (Herzig and Shaw, 2018). In addition, AMPK activation selectively regulate a subset of genes involved in the control of autophagy and lysosomal function, such as FOXO3, TFEB, and BRD4 (Li and Chen, 2019). Interestingly, results obtained in our lab suggested that oocytes from hyh mutant mice accumulate autophagic-like structures (de Paola et al., 2019). This observation is consistent with the function of α-SNAP in AMPK regulation (Wang and Brautigan, 2013) and in the maturation of autophagosomes (Abada et al., 2017). Similarly, Abada and collaborators demonstrated that the knockdown of α-SNAP leads to inhibition of autophagosome-lysosome fusion, leading to accumulation of autophagosomes (Abada et al., 2017). On the other hand, it has been proposed that loss of α-SNAP function induces non-canonical autophagy in human epithelia; however, the authors did not address whether this effect was AMPK-dependent (Naydenov et al., 2012b).

#### 4.3.4 Epigenetics

Finally, progression from NSPCs into differentiated neurons requires long-lasting changes in gene expression. Epigenetic mechanisms, including histone acetylation/deacetylation, DNA methylation, and non-coding RNA-mediated gene expression regulation, are essential during development. AMPK regulates diverse signaling networks that converge to epigenetically mediate transcriptional events (Gongol et al., 2018). AMPK enhances SIRT1 deacetylase activity, resulting in modulation of the activity of downstream SIRT1 targets (Canto et al., 2009). Combining whole-genome transcriptional and epigenetic analyses with *in vivo* functional studies, Bonnefont et al. nicely demonstrated that Bcl6, a transcriptional repressor that promotes neuronal differentiation of NSPCs, acts through Sirt1 recruitment followed by histone deacetylation and silencing of genes belonging to multiple pathways that promote self-renewal (Bonnefont et al., 2019). Interestingly, in other cell types such as follicular T cells, AMPK activation strongly induces Bcl6 upregulation and T cell differentiation (Xie et al., 2017).

Our findings indicate that the M105I mutation in ơ-SNAP leads to dysregulation of AMPK activity in NSPCs and premature neuronal differentiation both *in vitro* and *in vivo*. Furthermore, pharmacological inhibition of AMPK in hyh-NSPCs can prevent this abnormal cell fate commitment, strongly suggesting that, at least in part, AMPK dysregulation underlie the pathogenesis of hyh phenotype. Further investigation should focus on the mechanisms by which AMPK overactivation affects the biology of NSPCs.

## Data Availability

The original contributions presented in the study are included in the article/Supplementary Material, further inquiries can be directed to the corresponding authors.
