# Supplementary material for: Alpha-SNAP (M105I) mutation promotes neuronal differentiation of neural stem/progenitor cells through overactivation of AMPK
Source: Front Cell Dev Biol. 2023 Apr 11;11:1061777. doi: 10.3389/fcell.2023.1061777 (PMC10127105; doi:10.3389/fcell.2023.1061777)
Supplement: Supplementary file 1 [file Presentation1.pdf]

Supplementary Material

1 Supplementary Figures

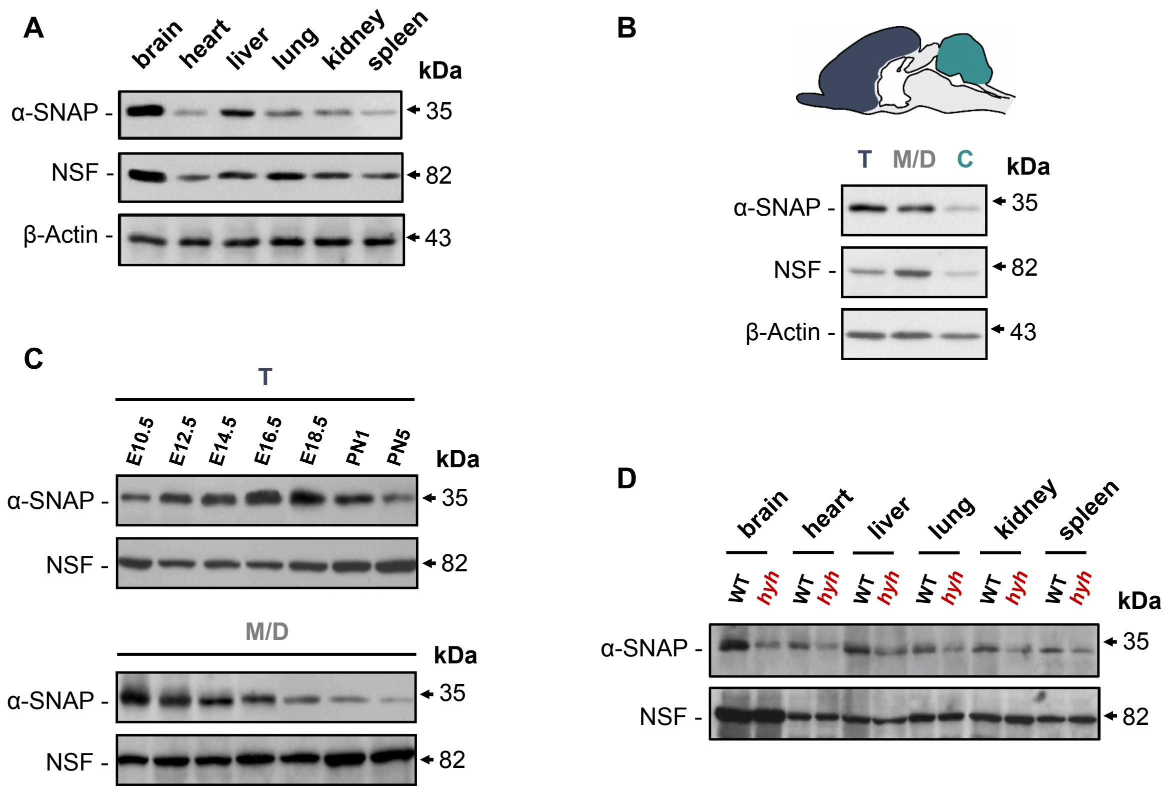

Supplementary Figure 1. α-SNAP expression and hypomorphism in diverse tissues of hyh mice. (A). Western blot analysis of α-SNAP and NSF protein levels in brain, heart, liver, lung, kidney and spleen of wild-type mice at postnatal day (PN) 1. **(B). Top:** Schematic representation of a wild type (WT) mouse brain at PN1. Telencephalon (blue, T), midbrain/diencephalon (grey, M/D) and cerebellum (green, C) are depicted. **Bottom:** Western blot analysis of α-SNAP and NSF in T, M/D and C protein homogenates (PN1). **(C).** Temporal expression pattern of α-SNAP and NSF in rostral (T) and caudal (M/D) regions of WT brain from E10.5 to PN5. **(D).** Western blot analysis of α-SNAP and NSF protein levels in brain, heart, liver, lung, kidney and spleen of wild-type and hyh mice at PN1. Note the hypomorphism of α-SNAP is conserved across different tissue samples.

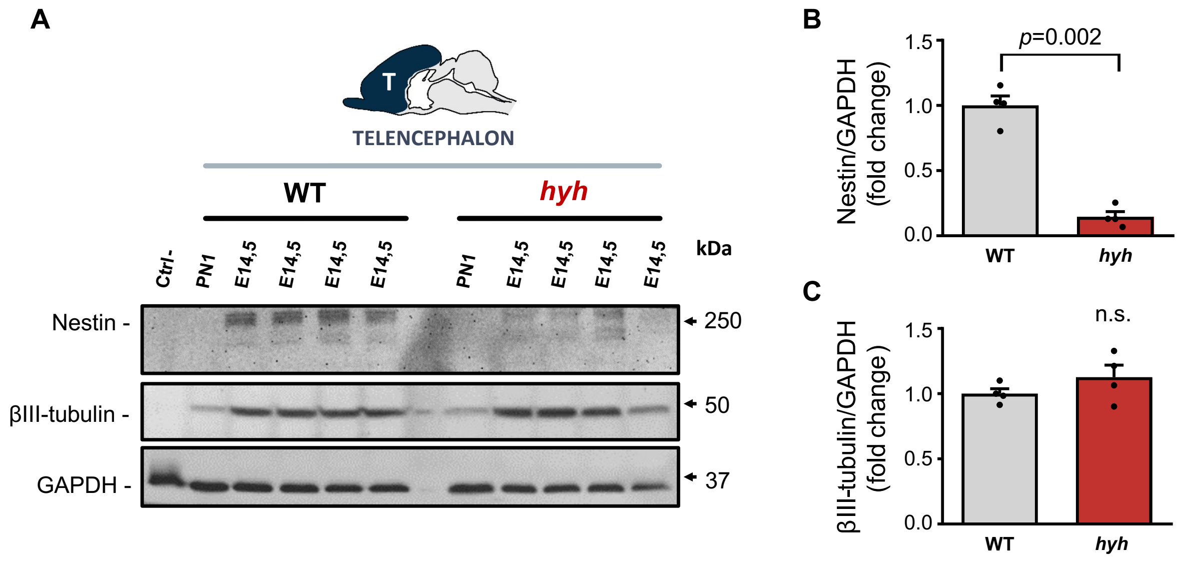

Supplementary Figure 2. Differentiation of NSPCs in the telencephalon of hyh mice. (A). Western blot analysis of Nestin and βIII-Tubulin protein levels in the telencephalon of wild-type and hyh mice at E14.5. **(B-C).** Densitometric analyzes of Nestin (B) and βIII-Tubulin (C). Differences with a p value <0.05 were considered statistically significant (Student t-test). n.s. = no significant differences.

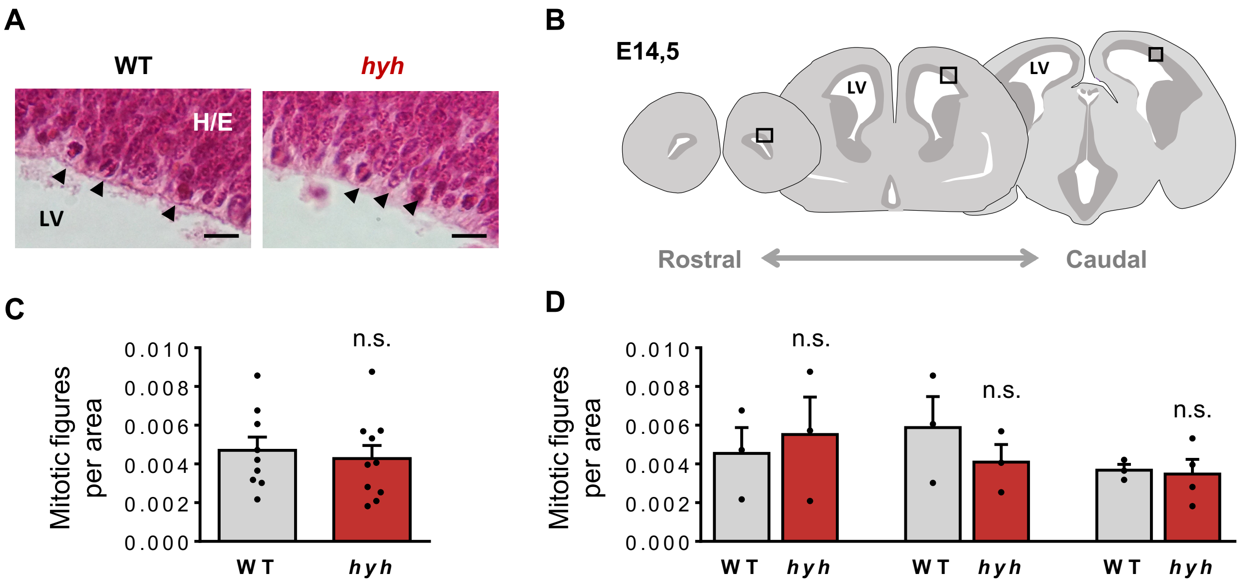

Supplementary Figure 3. Mitotic activity of NSPCs in the telencephalon of hyh mice. (A). Brain coronal sections of WT and hyh mice at E14.5 stained with hematoxylin/eosin (H&E). Black arrow heads indicate mitotic figures in ventricular lining cells. **(B).** Schematic representation of regions from rostral to caudal telencephalon in which the number of mitotic figures was determined. **(C).** Total number of mitotic figures in the VZ *per* area in WT and hyh mice. (**D).** Mitotic figures *per* area in WT and hyh mice in the rostral, medial and caudal dorsal wall of the telencephalon. Differences with a p value <0.05 were considered statistically significant (Student t-test). n.s. = no significant differences. *Scale Bar: A*= 20µm.

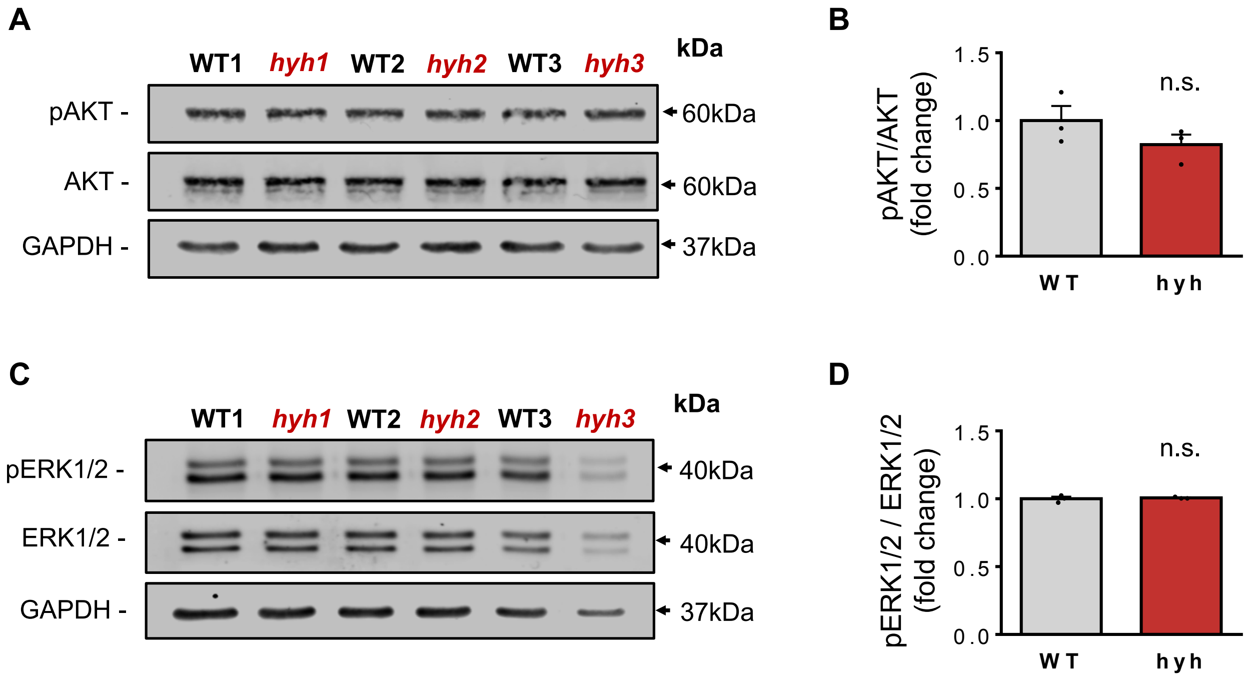

Supplementary Figure 4. Phosphorylation of AKT and ERK1/2 in NSPCs derived from hyh mice. (A, C). Western blot analysis of pAKT (A) and pERK1/2 (C) protein levels in neurospheres derived from WT and hyh mice. Biological replicates (triplicates) are shown. **(B, D).** Densitometric analysis of pAKT (B) and pERK1/2 (D). Differences with a p value <0.05 were considered statistically significant (Student t-test). n.s. = no significant differences.

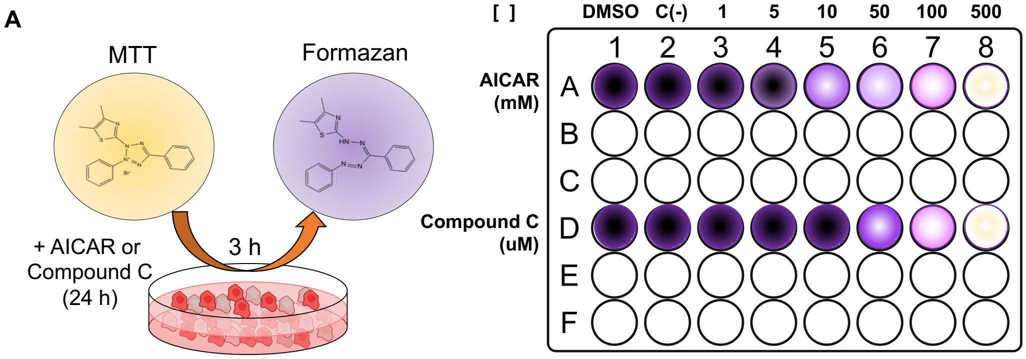

B

Absorbance

DMSO Control	Negative Control	1 mM	5 mM	10 mM	50 mM	100 mM	500 mM
0,838	0,948	0,804	0,528	0,248	0,142	0,027	0,034
0,901	0,914	0,846	0,672	0,391	0,205	0,065	0,019
0,747	0,889	0,784	0,584	0,447	0,099	0,072	0,025
0,987	1,089	0,941	0,526	0,421	0,168	0,121	0,009

C

Absorbance

DMSO Control	Negative Control	1 µM	5 µM	10 µM	50 µM	100 µM	500 µM
0,991	1,012	0,917	0,897	0,547	0,484	0,377	0,124
0,936	0,947	0,845	0,966	0,621	0,378	0,284	0,078
0,841	0,905	0,791	0,837	0,496	0,531	0,439	0,151
0,972	0,911	0,923	0,828	0,689	0,423	0,245	0,025

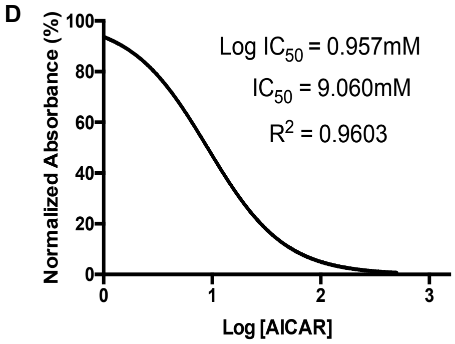

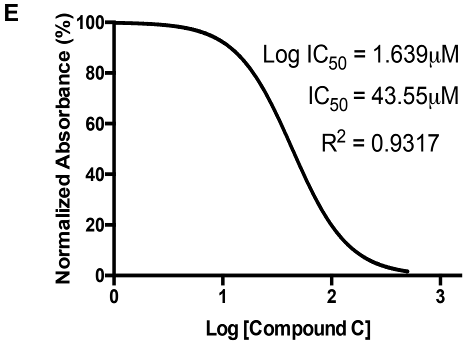

Supplementary Figure 5. Cell viability in response to pharmacological modulation of AMPK. (A). Scheme of the experimental design to determine the cytotoxicity of AICAR and Compound C based on the oxidation of MTT to formazan. **(B-C).** Absorbance values obtained by spectrophotometry. **(D).** Representative curve of absorbance values in response to AICAR treatment, considering the following doses: 1 mM, 5 mM, 10 mM, 50 mM, 100 mM and 500 mM. **(E).** Representative curve of the absorbance values in response to treatment with Compound C, considering the following doses: 1 µM, 5 µM, 10 µM, 50 µM, 100 µM and 500 µM. Note, the IC50 for AICAR and Compound C was 9.06 mM and 43.55 µM, respectively. Incubation with no drugs (negative control; C(-)) or with solvent (dimethyl sulfoxide; DMSO) were used as controls. Absorbance was normalized from control MEFs.

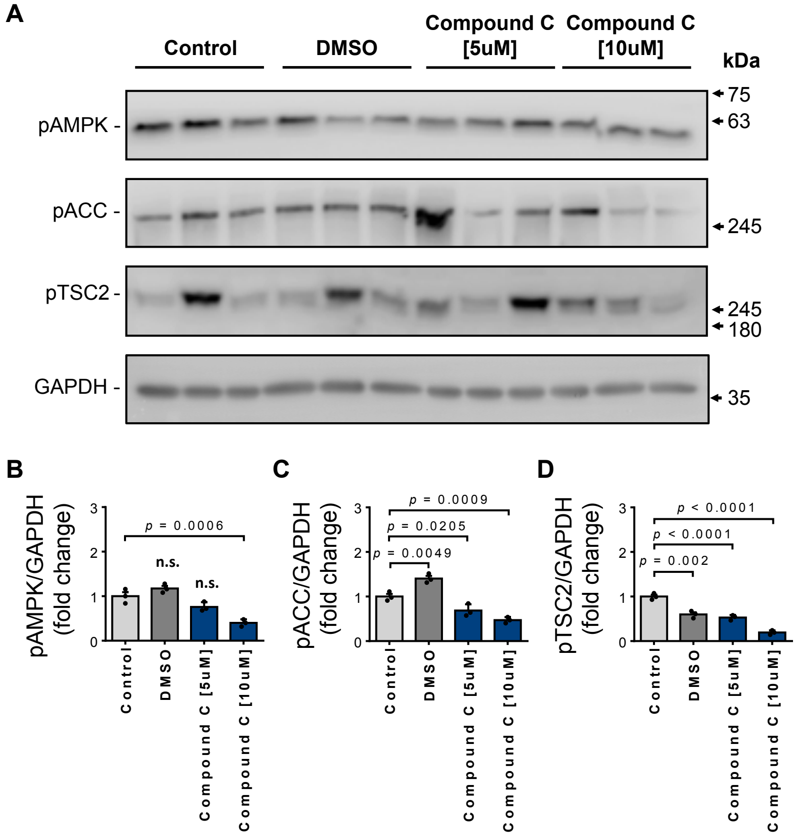

Supplementary Figure 6. Pharmacological modulation via Compound C induces significant changes in the phosphorylation of AMPK and its downstream targets. (A). Western blot analysis of pAMPK, pACC, and pTSC2 protein levels in MEFs treated with 5 µM and 10 µM of Compound C. **(B-D).** Densitometric analysis of pAMPK (B), pACC (C), and pTSC2 (D). Note the inhibition of AMPK correlates with a decrease in the phosphorylation of pACC and pTSC2. Differences with a p value <0.05 were considered statistically significant (One-way ANOVA, multiple comparison). n.s. = no significant differences.

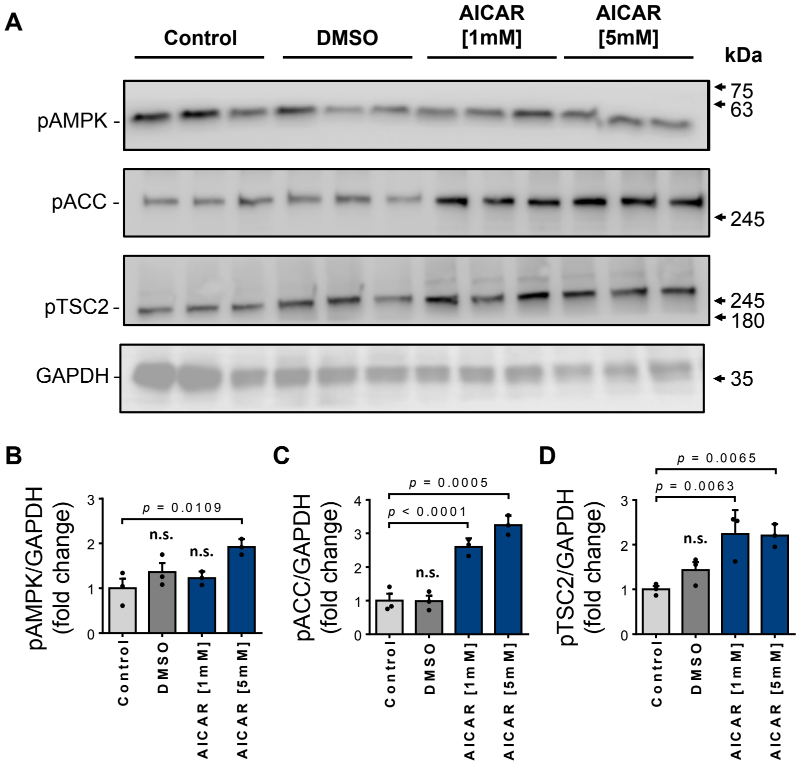

Supplementary Figure 7. Pharmacological modulation via AICAR induces significant changes in the phosphorylation of AMPK and its downstream targets. (A). Western blot analysis of pAMPK, pACC, and pTSC2 protein levels in MEFs treated with 1 mM and 5 mM of AICAR. **(B-D).** Densitometric analysis of pAMPK (B), pACC (C), and pTSC2 (D). Note the activation of AMPK correlates with an increase in the phosphorylation of pACC and pTSC2. Differences with a p value <0.05 were considered statistically significant (One-way ANOVA, multiple comparison). n.s. = no significant differences.

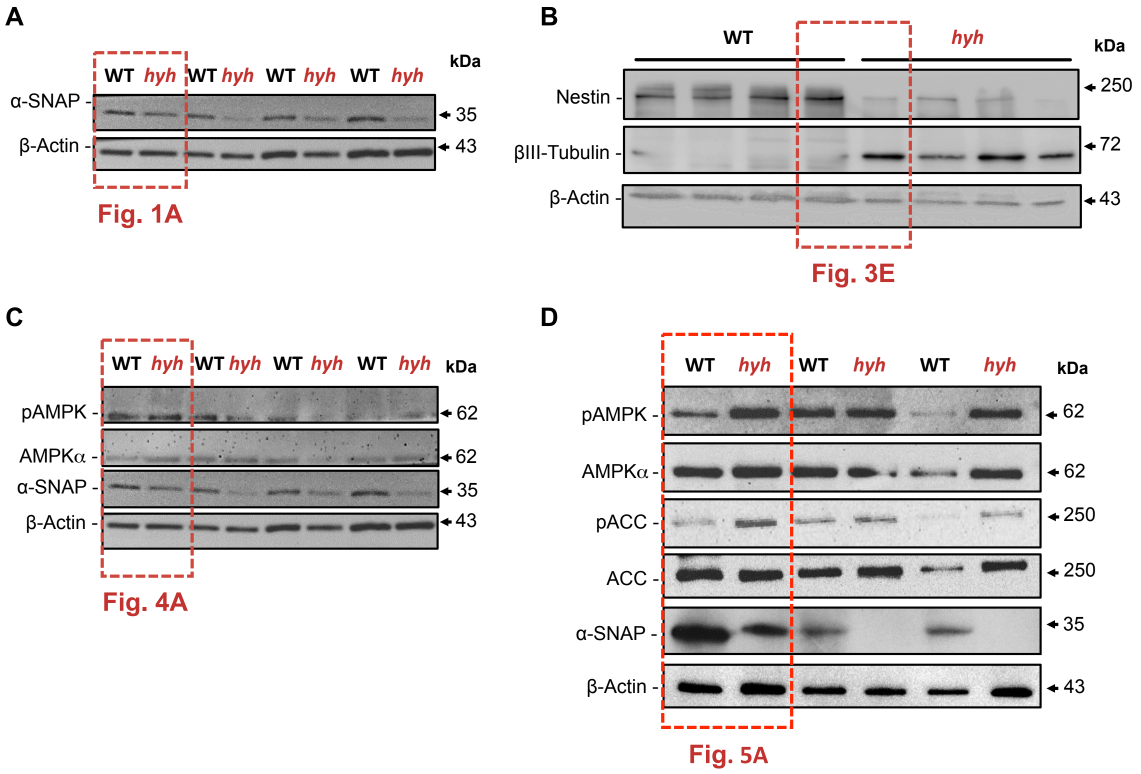

Supplementary Figure 8. Biological replicates of Western blot studies. (A). Western blot analysis of WT and hyh telencephalic lysates at E14,5 using antibodies against α-SNAP. β-actin was used as loading control. The red square depicts the lanes used in figure 1A. **(B).** Western blot analysis of WT and hyh neurosphere (NS) lysates using antibodies against Nestin and βIII-tubulin. β-actin was used as loading control. The red square depicts the lanes used in figure 3E. **(C).** Western blot analysis of proteins obtained from WT and hyh telencephalon at E14,5 using antibodies against α-SNAP, pAMPK and total AMPKα. The red square depicts the lanes used in figure 4A. **(D).** Western blot analysis of 7 DIV NS obtained from WT and hyh mice using antibodies against α-SNAP, pAMPK, AMPKα, pACC and ACC. The red square depicts the lanes used in figure 5A.
